# Taxonomic revision of the genus *Carasobarbus* Karaman, 1971 (Actinopterygii, Cyprinidae)

**DOI:** 10.3897/zookeys.339.4903

**Published:** 2013-10-03

**Authors:** Kai Borkenhagen, Friedhelm Krupp

**Affiliations:** 1 Senckenberg Research Institute and Museum of Nature, Senckenberganlage 25, 60325 Frankfurt am Main, Germany; 2 Research and Technology Centre (FTZ), University of Kiel, Hafentörn 1, D-25761 Büsum, Germany; 3 Qatar Museums Authority, P.O. Box 2777, Doha, Qatar

**Keywords:** Cyprinidae, SW Asia, NW Africa, taxonomy

## Abstract

Representatives of the fish genus *Carasobarbus* Karaman, 1971 (Actinopterygii: Cyprinidae) from the Middle East and North Africa were previously placed in 14 different genus-group taxa (*Barbellion*, *Barbus*, *Barynotus*, *Capoeta*, *Carasobarbus*, *Cyclocheilichthys*, *Kosswigobarbus*, *Labeobarbus*, *Luciobarbus*, *Pseudotor*, *Puntius*, *Systomus*, *Tor* and *Varicorhinus*). The generic assignment of several species changed frequently, necessitating a re-evaluation of their taxonomic status. In this study, the genus *Carasobarbus* is revised based on comparative morphological examinations of about 1300 preserved specimens from collections of several museums and freshly collected material. The species *Carasobarbus apoensis*, *Carasobarbus canis*, *Carasobarbus chantrei*, *Carasobarbus exulatus*, *Carasobarbus fritschii*, *Carasobarbus harterti*, *Carasobarbus kosswigi*, *Carasobarbus luteus* and *Carasobarbus sublimus* form a monophyletic group that shares the following combination of characters: medium-sized barbels with a smooth last unbranched dorsal-fin ray, nine or 10 branched dorsal-fin rays and six branched anal fin-rays; scales large, shield-shaped, with many parallel radii; the lateral line containing 25 to 39 scales; the pharyngeal teeth hooked, 2.3.5-5.3.2 or 2.3.4-4.3.2; one or two pairs of barbels. The species are described in detail, their taxonomic status is re-evaluated and an identification key is provided. A lectotype of *Systomus luteus* Heckel, 1843 is designated. *Carasobarbus* Karaman, 1971, *Kosswigobarbus* Karaman, 1971, and *Pseudotor* Karaman, 1971 are subjective synonyms, and acting as First Reviser we gave precedence to the name *Carasobarbus*.

## Introduction

The species of the cyprinid genus *Carasobarbus* Karaman, 1971 are distributed across SW Asia and NW Africa. They occur in all major river systems of the Levant, Mesopotamia, southern Iran, the western and south-western Arabian Peninsula and in northern Morocco. *Carasobarbus* species are an important element of the ichthyofaunas of these areas.

Research about the Mesopotamian and Levantine representatives of *Carasobarbus* began as early as the middle of the 19^th^ century. Important ichthyologists of that era, such as A. Valenciennes and J. J. Heckel, were the first to study these fish. One of the most prominent biological collections from the Middle East of this time was made by T. Kotschy from 1836 to 1840. It is stored at the Museum of Natural History of Vienna and encompasses the type specimens of many zoological and botanical taxa (Kähsbauer 1963). The Moroccan ichthyofauna was described towards the end of the 19^th^ century and the start of the 20^th^ century by A. Günther and G. A. Boulenger. An expedition lead by C. du Gast in 1912 was one of the first systematic sampling efforts in this area (Pellegrin 1912). The Arabian representatives were reported only about 35 years ago by K. E. Banister and M. A. Clarke. In 1971, M.S. Karaman established the monotypic genus *Carasobarbus* for *Systomus luteus* Heckel, 1843 characterised by a laterally compressed and high-backed body, a narrow and high head, a single pair of barbels, pharyngeal bones with three rows of spoon-shaped teeth, a pharyngeal teeth count 2.3.5-5.3.2, a subterminal to terminal mouth, weakly developed lips that run along the jaws as a thin band, no median lobe on the lower lip, infraorbital bones of normal size, a short and broad first infraorbital (lacrimal) bone that is shorter than the eye diameter, a dorsal fin that is moderately long and has 10 branched rays, the origin of the dorsal fin being above the ventral fins, the last unbranched ray of the dorsal fin being well ossified and smooth, the anal fin with six branched rays, and large scales with numerous parallel radii. We revised and expanded Karaman’s (1971) diagnosis of the genus that now contains the nine following species: *Carasobarbus apoensis* (Banister & Clarke, 1977), *Carasobarbus canis* (Valenciennes in Cuvier and Valenciennes 1842), *Carasobarbus chantrei* (Sauvage, 1882), *Carasobarbus exulatus* (Banister & Clarke, 1977), *Carasobarbus fritschii* (Günther, 1874), *Carasobarbus harterti* (Günther, 1901), *Carasobarbus kosswigi* (Ladiges, 1960), *Carasobarbus luteus* (Heckel, 1843), and *Carasobarbus sublimus* (Coad & Najafpour, 1997). Members of this genus were listed in 14 different genera and subgenera in the past: *Barbellion* Whitley, 1931, *Barbus* Cuvier, 1816, *Barynotus* Günther, 1868, *Capoeta* Valenciennes in Cuvier and Valenciennes 1842, *Carasobarbus* Karaman, 1971, *Cyclocheilichthys* Bleeker, 1859, *Kosswigobarbus* Karaman, 1971, *Labeobarbus* Rüppell, 1835, *Luciobarbus* Heckel, 1843, *Pseudotor* Karaman, 1971, *Puntius* Hamilton, 1822, *Systomus* McClelland, 1839, *Tor* Gray, 1834, and *Varicorhinus* Rüppell, 1835.

The objectives of the current study are to (1) define a monophyletic genus that is based on synapomorphic characters, (2) provide a conclusive diagnosis of the genus *Carasobarbus*, (3) give a detailed re-description of all species based on a sample of specimens large enough to show the intraspecific variability, (4) map the range of each species based on records confirmed by voucher specimens, (5) summarise information on biology, habitat and conservation status of each species, (6) discuss the taxonomic history and current status of each species, (7) provide an identification key. This will form a baseline for a molecular phylogenetic and zoogeographic analysis of *Carasobarbus* and related genera that is currently in preparation and will be published separately by the first author.

## Methods

Abbreviations for ichthyological collections follow Sabaj-Pérez (2010) and Fricke and Eschmeyer (2013).

Nomenclature of geographic names follows the spelling recommended by the “United States Board on Geographic Names” (http://geonames.usgs.gov/), even though the transcriptions/transliterations of these toponyms are sometimes inconsistent. Wādī or Oued refer to a temporary stream. Nahr, Naẖal, Nehri, Rūdkhāneh or Rūd refer to a permanent river or stream. Buḩayratt, Göl or Daryācheh refer to a lake. ‘Ayn or Aïn refer to a spring. Geographical coordinates in parentheses are original coordinates, given by a publication, the collector or a collection database. Coordinates determined ex post are marked by brackets. Most of these are from the National Geospatial-Intelligence Agency gazetteer (http://geonames.nga.mil/ggmagaz/) and as a consequence, do not refer to the actual site of collection, but to the geographic feature itself. For some of the well known waterbodies and cities the conventional name is used: Euphrates (Nahr al Furāt / Fırat Nehri), Jordan River (HaYarden / Nahr al Urdan), Lake Homs (Buḩayratt Qaţţīnah), Lake Tiberias (Yam Kinneret / Buḩhayratt Ţabarīyā), Orontes (Nahr al ‘Āşī / Asi Nehri), Tigris (Dicle Nehri / Nahr Dijlah), Aleppo (Ḩalab), Damascus (Dimashq), Mosul (Al Mawşil).

Twenty morphometric measurements were taken from specimens straightened whenever necessary; severely damaged and bent specimens were not used. There are some differences in the way of taking measurements (e.g. Hubbs and Lagler 1958 vs. Banister and Clarke 1977, Krupp 1983a). In this study, seven measurements were done over projections to the body axis. They are as follows: total length (the distance between projections of the tip of the snout and the posterior margin of the longest lobe of the caudal fin, with the caudal fin spread to its natural maximum), standard length (SL) (the distance between projections of the tip of the snout and the end of the hypural plate), preanal length (the distance between projections of the tip of the snout and the origin of the anal fin), predorsal length (the distance between projections of the tip of the snout and the origin of the dorsal fin), preventral length (the distance between projections of the tip of the snout and the origin of the ventral fin), head length (HL) (the distance between projections of the tip of the snout and the posterior margin of the bony opercle), and length of the caudal peduncle (the distance between projections of the insertion of the anal-fin base and the end of the hypural plate). The other measurements were done point-to-point: body depth (BD) as the maximum depth of the body (without dorsal fin) taken orthogonal to body axis; depth (minimum) of the caudal peduncle as the smallest depth of the caudal peduncle; length of the dorsal and anal fins as length of the last unbranched ray in the dorsal and anal fins, respectively; lengths of the pectoral and ventral fins as the distance from the fin base to the tip of the pectoral and ventral fins, respectively; length of the dorsal-fin base and the anal-fin base as a distance between the origin and the insertion of the fin; length of the anterior and posterior barbels as distance from the barbel base to the tip of the barbel when straightened; horizontal diameter of the eye as a distance between the anterior and the posterior bony margins of the eye cavity; width of the mouth as a distance between the two posterior ends of the lower jaw; interorbital distance as a distance between the upper margins of the eye cavities across the head. For comparison between species, all measurements are expressed as percentage of SL. To attenuate effects of allometric growth, only specimens in the size range between 50 mm SL and 150 mm SL were used for the box-plots and the data given in Table 1.

**Table 1.** Comparison of morphometric characters of specimens between 50 mm SL and 150 mm SL. All measurements expressed as percentage of SL.

		**total length**	**preanal length**	**predorsal length**	**preventral length**	**head length**	**caudal peduncle length**	**body depth**	**caudal peduncle depth**	**dorsal fin length**	**pectoral fin length**	**ventral fin length**	**anal fin length**	**dorsal-fin base length**	**anal-fin base length**	**anterior barbel length**	**posterior barbel length**	**eye diameter**	**mouth width**	**interorbital distance**
*Carasobarbus apoensis*	max	130.5	81.0	55.8	57.9	31.2	18.1	32.6	12.3	24.5	22.6	20.0	21.3	19.4	10.8	0.0	6.6	8.1	9.0	10.5
	min	121.0	76.3	48.5	51.3	26.5	11.8	25.4	10.2	16.7	18.1	16.9	16.8	15.1	7.2	0.0	2.4	5.5	5.7	8.1
	med	126.0	78.8	53.1	53.9	28.8	14.2	29.1	11.1	21.1	20.2	18.1	18.8	17.3	8.8	0.0	4.8	6.3	7.4	9.5
	n	41	43	44	44	44	44	44	44	42	44	44	43	44	44	0	44	44	44	44
*Carasobarbus canis*	max	132.6	82.1	56.4	58.0	32.4	17.2	31.2	12.6	30.7	24.4	21.9	22.3	20.5	10.1	3.9	6.3	9.0	8.8	9.6
	min	121.2	75.0	47.8	50.9	26.2	12.5	26.7	9.9	18.0	18.4	16.1	14.9	16.8	7.0	0.7	2.3	5.4	6.2	7.1
	med	126.3	78.4	51.6	54.3	29.1	14.8	28.9	11.9	21.5	20.5	18.0	18.7	18.6	8.6	2.3	4.8	6.6	7.1	8.6
	n	54	56	56	56	56	56	56	56	46	56	56	56	56	56	52	55	56	54	56
*Carasobarbus chantrei*	max	134.8	82.3	53.9	55.3	30.0	17.1	35.8	14.0	27.5	24.4	21.8	23.8	21.2	10.2	2.5	4.7	8.9	8.1	10.3
	min	121.9	72.8	47.7	50.6	22.2	11.7	26.4	11.0	18.8	17.6	16.4	16.3	17.2	7.0	0.5	2.2	5.0	5.8	7.7
	med	129.7	77.9	50.6	53.0	26.1	14.5	30.8	12.6	24.6	21.7	19.8	20.4	19.2	8.8	1.1	3.4	6.9	6.9	9.2
	n	81	84	84	84	84	84	84	84	76	82	84	83	84	84	68	84	84	81	84
*Carasobarbus exulatus*	max	134.8	82.3	53.9	55.3	30.0	17.1	35.8	14.0	27.5	24.4	21.8	23.8	21.2	10.2	2.5	4.7	8.9	8.1	10.3
	min	121.9	72.8	47.7	50.6	22.2	11.7	26.4	11.0	18.8	17.6	16.4	16.3	17.2	7.0	0.5	2.2	5.0	5.8	7.7
	med	129.7	77.9	50.6	53.0	26.1	14.5	30.8	12.6	24.6	21.7	19.8	20.4	19.2	8.8	1.1	3.4	6.9	6.9	9.2
	n	81	84	84	84	84	84	84	84	76	82	84	83	84	84	68	84	84	81	84
*Carasobarbus fritschii*	max	137.5	82.9	53.7	55.3	26.8	18.8	34.3	13.6	28.0	25.2	23.8	31.3	20.6	11.9	5.6	8.3	8.8	9.8	10.7
	min	121.1	73.6	44.9	47.0	18.8	10.7	24.8	10.6	19.6	19.3	15.8	18.1	15.0	6.6	1.4	2.8	4.7	5.4	6.3
	med	129.8	77.1	49.3	50.7	23.0	15.0	29.2	11.9	23.6	22.2	20.5	22.4	17.4	9.1	3.1	4.7	6.6	7.1	9.1
	n	229	243	244	243	244	244	244	243	196	244	244	242	243	243	242	244	244	244	244
*Carasobarbus harterti*	max	140.9	78.0	53.7	55.8	27.5	18.4	31.6	13.3	31.8	25.5	24.8	24.1	19.0	10.3	9.1	9.6	9.5	7.1	9.6
	min	122.6	70.9	46.5	48.5	21.2	12.3	26.8	11.8	25.8	21.5	20.4	18.9	16.5	8.4	4.5	5.5	5.9	5.2	7.4
	med	131.8	75.0	49.8	51.1	24.4	16.0	29.2	12.8	28.9	23.5	22.8	21.1	18.0	9.3	6.6	7.8	7.4	6.2	8.4
	n	19	24	24	24	24	24	24	24	11	24	23	23	24	24	24	24	24	24	24
*Carasobarbus kosswigi*	max	133.9	79.7	53.4	51.9	25.3	16.0	32.8	12.9	35.5	23.8	21.8	26.4	21.8	11.1	5.5	7.4	8.1	5.9	8.5
	min	127.1	75.4	47.0	48.7	22.8	11.9	26.2	10.4	26.1	20.1	19.1	20.0	18.1	8.8	2.8	3.6	4.8	3.7	7.3
	med	130.4	77.7	49.7	50.8	24.5	14.5	31.1	11.9	28.8	22.1	20.8	22.6	19.9	10.0	4.3	5.1	5.9	4.6	7.9
	n	14	15	15	15	15	15	15	15	14	15	15	15	15	15	15	15	15	15	15
*Carasobarbus luteus*	max	134.1	84.0	56.3	57.9	33.1	15.8	40.1	14.3	31.9	24.6	22.7	23.7	22.6	10.7	3.2	7.1	10.1	10.7	11.3
	min	120.4	74.7	47.3	48.6	21.7	8.6	26.2	11.0	17.6	17.9	16.8	15.8	14.9	6.6	0.6	2.8	0.0	5.1	7.4
	med	127.5	79.1	52.2	53.4	27.3	13.2	33.6	12.8	24.9	21.4	19.8	19.9	19.2	9.0	1.7	4.4	7.2	7.1	9.6
	n	257	264	268	265	267	266	268	268	241	267	268	266	267	267	41	266	265	233	268
*Carasobarbus sublimus*	max	137.9	81.4	57.0	56.8	30.1	14.8	33.4	13.8	29.9	25.5	23.8	28.4	22.1	11.5	7.0	9.7	10.0	7.6	9.0
	min	131.9	76.5	49.1	49.6	25.2	10.3	27.9	11.8	19.7	22.9	21.0	21.9	19.4	8.7	4.1	5.1	5.6	3.6	6.8
	med	134.4	77.9	52.5	54.2	27.7	13.0	30.6	12.8	28.3	24.3	22.2	23.9	20.7	10.2	5.4	8.0	8.9	6.4	8.4
	n	16	18	18	18	18	18	18	18	14	18	18	18	18	18	18	18	18	18	18

In addition, seven meristic characters were analysed. The last branched anal- and dorsal-fin rays were counted as one when lying directly adjacent to each other without an interspace. Scales in the lateral line were counted from the first scale with a pore to the last scale on the caudal peduncle (some authors only count to the end of the hypural plate). Scales above the lateral line were counted between the origin of the dorsal fin and the lateral line; the lateral line was not included and a scale on the mid-line of the back was counted as 0.5. Scales below the lateral line were counted between origin of the anal fin to the lateral line; the lateral line was not included and a scale on the mid-line of the belly was counted as 0.5. Scales around the least circumference of the caudal peduncle were counted as one circle of scales around the least circumference of the caudal peduncle. Number of pairs of barbels was counted as two when anterior and posterior pairs are present, counted as one if only posterior pair is present and counted as 1.5 if posterior pair and one single anterior barbel is present.

For counting the number of pharyngeal teeth, the pharyngeal bones were extracted in a subsample of specimens and the pharyngeal teeth counted sometimes only on one side. Lost teeth were counted when the point of insertion was clearly visible. Scales were extracted in the anterior part of the body, above the lateral line.

We did not differentiate between male and female specimens because sex determination was not possible without dissection.

## Results

### Genus Carasobarbus

Karaman, 1971

http://species-id.net/wiki/Carasobarbus

Carasobarbus Karaman 1971: 230; type species: *Systomus luteus* Heckel, 1843, by original designation, also by monotypy.
Kosswigobarbus Karaman 1971: 239; type species: *Cyclocheilichthys kosswigi* Ladiges, 1960, by original designation, also by monotypy.
Pseudotor Karaman 1971: 229; type species: *Barbus fritschii* Günther, 1874, by original designation.

#### Diagnosis.

Medium-sized cyprinids with an ossified, smooth last unbranched dorsal-fin ray; 9 or 10 branched dorsal-fin rays and 6 branched anal-fin rays; large, shield-shaped scales with numerous parallel radii; the lateral line with 25 to 39 scales; the pharyngeal teeth hooked at their tips, their count being 2.3.5-5.3.2 or 2.3.4-4.3.2; 1 or 2 pairs of barbels present.

*Carasobarbus* species are evolutionarily hexaploid (Machordom and Doadrio 2001, Gorshkova et al. 2002, Leggatt and Iwama 2003, Tsigenopoulos et al. 2010).

#### Remarks and discussion.

‘*Barbus*’ *grypus*, *Mesopotamichthys sharpeyi* and ‘*Barbus*’ *reinii* from the Middle East have five branched rays in the anal fin. The hexaploid species from Africa (*Labeobarbus* and *Varicorhinus*), which are the sister group to *Carasobarbus* and the other species from the Middle East (Tsigenopoulos et al. 2010, KB unpublished), have five branched rays in the anal fin. The Asian species (*Tor* and *Neolissochilus*) are sister group to the species from Africa and the Middle East (Tsigenopoulos et al. 2010, KB unpublished) and have five branched rays in the anal fin. By application of the parsimony principle the possession of six branched anal-fin rays is a synapomorphy of the genus *Carasobarbus*. The possession of nine to 10 branched rays in the dorsal fin is synapomorphic for *Carasobarbus*, because the closely related Middle-Eastern species ‘*Barbus*’ *grypus*, *Mesopotamichthys sharpeyi* and ‘*Barbus*’ *reinii* as well as many African hexaploids have the plesiomorphic state of eight branched rays in the dorsal fin. However, in some African species the number of branched dorsal-fin rays is increased convergently. These two synapomorphies establish *Carasobarbus* as a monophyletic group. Analyses of the mitochondrial cytochrome *b* gene confirm the monophyly of this genus (Durand et al. 2002, Tsigenopoulos et al. 2010, KB unpublished data). Colli et al. (2009) found *Carasobarbus* to be monophyletic in their maximum likelihood analysis but not in their neighbour joining analysis. ‘*Barbus*’ *grypus* Heckel, 1843 is the sister taxon of the genus *Carasobarbus* (Tsigenopoulos et al. 2010).

Out of the generic names *Barbellion*, *Barbus*, *Barynotus*, *Capoeta*, *Carasobarbus*, *Cyclocheilichthys*, *Kosswigobarbus*, *Labeobarbus*, *Luciobarbus*, *Pseudotor*, *Puntius*, *Systomus*, *Tor*, and *Varicorhinus* that were used for this taxon − or its parts − by previous authors, only *Carasobarbus*, *Kosswigobarbus* and *Pseudotor* are available for the genus in question. All other generic names have not been considered, because their type species are not closely related to the species under discussion here (Durand et al. 2002, Tsigenopoulos et al. 2010, KB unpublished data) or do not share the characters mentioned above. *Carasobarbus*, *Kosswigobarbus* and *Pseudotor* are subjective synonyms. They all were established in the same publication (Karaman 1971) and thus none of them has priority. We, acting as First Reviser, select *Carasobarbus* to have priority in accordance with article 24.2 of the International Code for Zoological Nomenclature (ICZN 2012). Thus *Carasobarbus* is the valid name for this genus.

Within the genus, several species share characters that are potentially synapomorph and elucidate sister group relations. *Carasobarbus fritschii* and *Carasobarbus harterti* both have pharyngeal bones with four teeth in the medial row. This character is probably synapomorph, because all other congeners have five teeth in the medial row. This group corresponds to *Pseudotor*. *Carasobarbus kosswigi* and *Carasobarbus sublimus* share the possession of a spatulate lower jaw and a median lobe on the lower lip. The spatulate lower jaw is synapomorph, because no congener and no other closely related species shares this character. The close phylogenetic relationship between *Carasobarbus kosswigi* and *Carasobarbus sublimus* is confirmed by genetic analysis (Borkenhagen et al. 2011). These two species correspond to *Kosswigobarbus*.

### Carasobarbus apoensis

(Banister & Clarke, 1977)

http://species-id.net/wiki/Carasobarbus_apoensis

Barbus apoensis Banister and Clarke 1977: 113.

#### Material.

**Type material.** Holotype of *Barbus apoensis*: BMNH 1976.4.7:166, Saudi Arabia, permanent stream near Khamīs Mushayt (18°17'N, 42°34'E), F. Tippler, 12 Dec 1968.

Paratypes of *Barbus apoensis*: BMNH 1976.4.7:167-171, 5, same data as holotype. - BMNH 1976.4.7:172-175, 4, Saudi Arabia, upper Wādī Turabah near Aţ Ţā’if (22°56'N, 40°54'E), G. Popov. - BMNH 1971.2.11:1-2, 2, Saudi Arabia, intermittent watercourse in Wādī Adamah (19°53'N, 41°57'E), J. P. Mandaville, 27 Oct 1969.

**Non-type material.** Endorheic darinages. BMNH 1980.7.1:15, 1, Saudi Arabia, Wādī Habayaba between Aţ Ţā’if and Ash Shafā [N21°11’, E 40°24'], A. Farag, 1980. - SMF 30167, 3; SMF 30170, 10 Saudi Arabia, Wādī Būwah (20°45'N, 41°8'E), F. Krupp and W. Schneider, 21 Mar 1990. - SMF 30169, 6; SMF 33147, 4, Saudi Arabia, Wādī Būwah (20°44'N, 41°7'E), F. Krupp and W. Schneider, 21 Mar 1990. - SMF 30168, 6; SMF 30171, 9, Saudi Arabia, Wādī Turabah (20°32'N, 41°17'E), F. Krupp and W. Schneider, 20 Mar 1990.

Streams draining towards the Red Sea. CMNFI 87-0135, 1; CMNFI 87-0137, 4, Saudi Arabia, Wādī Hadīyah (25°34'N, 38°41'E). - SMF 33149, 1, Saudi Arabia, Wādī Ḩaqqaq (22°49'N, 39°22'E), W. Büttiker, 5/6 May 1983. - SMF 33148, 2, Saudi Arabia, Wādī ‘Ilyab (20°5'N, 40°54'E), H. Felemban and J. Gasparetti, 28 Oct 1983. - SMF 33539, 3, Saudi Arabia, Wādī ‘Ilyab (20°7'N, 40°57E), W. Büttiker, 10−11 Nov 1983.

Unknown drainage system. SMF 33146, 4, Saudi Arabia, Al Ḩijāz, W. Büttiker.

#### Diagnosis.

One pair of barbels, usually 10 branched rays in the dorsal fin, 27 to 32 scales in the lateral line, usually 12 scales around the least circumference of the caudal peduncle, last unbranched ray of dorsal fin shorter than head.

#### Description.

**Figure 1.**
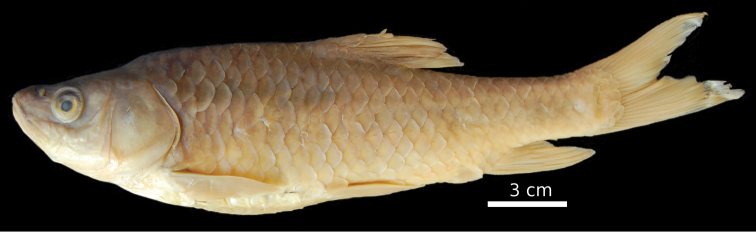
*Carasobarbus apoensis*, holotype (BMNH 1976.4.7:166) from a permanent stream near Khamīs Mushayt, ^©^ The Natural History Museum, London.

**Figure 2.**
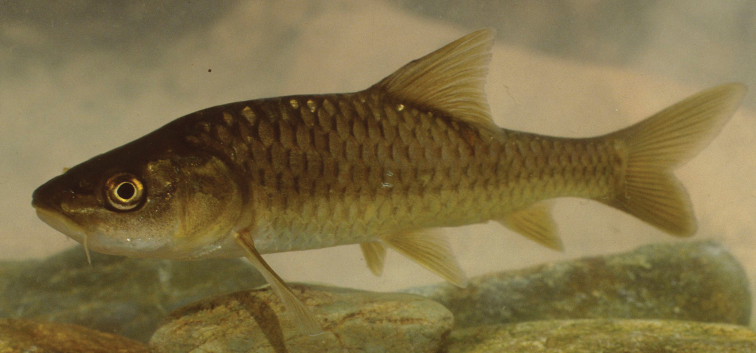
*Carasobarbus apoensis*, live specimen from Wādī Turabah.

**Figure 3.**
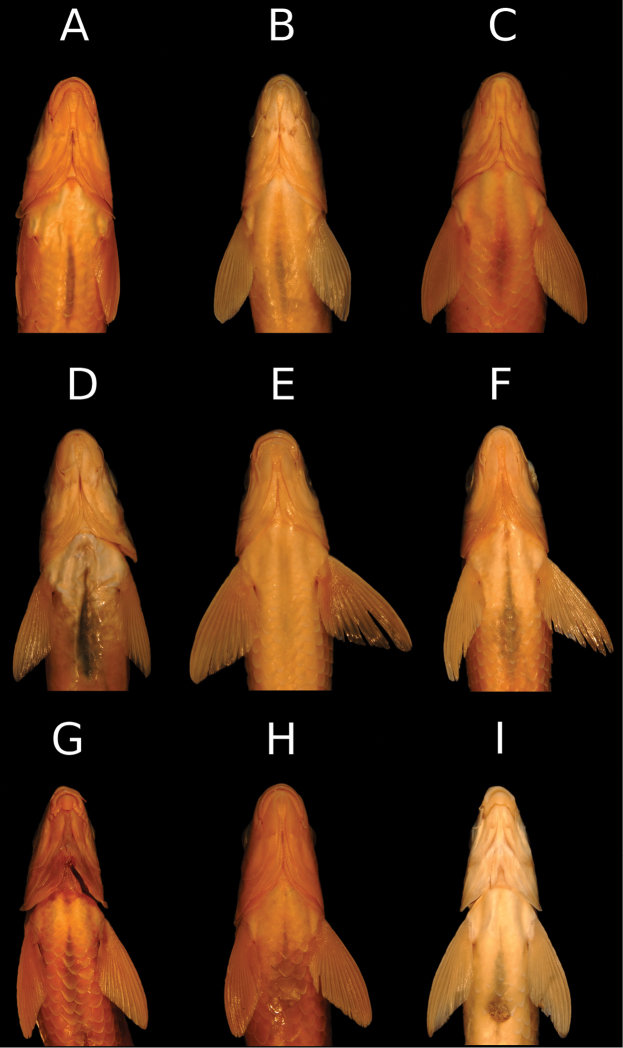
Ventral view of the head and chest. **A** *Carasobarbus apoensis* (SMF 30167, 108.6 mm SL) **B** *Carasobarbus canis* (SMF 33135, 108.3 mm SL) **C** *Carasobarbus chantrei* (SMF 33133, 122.9 mm SL) **D** *Carasobarbus exulatus* (SMF 33109, 103.7 mm SL) **E** *Carasobarbus fritschii* (SMF 33446, 89.6 mm SL) **F** *Carasobarbus harterti* (SMF 33368, 93.6 mm SL) **G** *Carasobarbus kosswigi* (SMF 30173, 107.1 mm SL) **H** *Carasobarbus luteus* (SMF 30176, 120.7 mm SL) **I** *Carasobarbus sublimus* (SMF 33118, 80.2 mm SL), pictures resized to facilitate comparison.

The body depth is comparatively low and a nuchal hump is present in adults but not developed in juveniles. The height of the caudal peduncle is relatively low (Table 1). The dorsal and ventral fins are usually positioned behind the middle of the body. The head is elongate with a straight or slightly concave dorsal profile. The ventral profile of the head is slightly convex. (Figs 1, 2). The head length is about equal to the body depth. The mouth is broad and terminal or slightly sub-terminal with one pair of barbels (Fig. 3, Table 2). Only one out of 65 specimens had two pairs of barbels and in one specimen a single anterior barbel was present. The eyes are in the anterior half of the head and slightly protuberant. The morphometric characters are summarised in Table 1.

**Table 2.** Number of pairs of barbels.

	**n**	**1**	**1,5**	**2**
*Carasobarbus apoensis*	65	63	1	1
*Carasobarbus canis*	89	4	1	84
*Carasobarbus chantrei*	157	5	6	146
*Carasobarbus exulatus*	83			83
*Carasobarbus fritschii*	299	2		297
*Carasobarbus harterti*	30			30
*Carasobarbus kosswigi*	23			23
*Carasobarbus luteus*	421	365	9	47
Naband population	10			10
*Carasobarbus sublimus*	18			18

**Figure 4.**
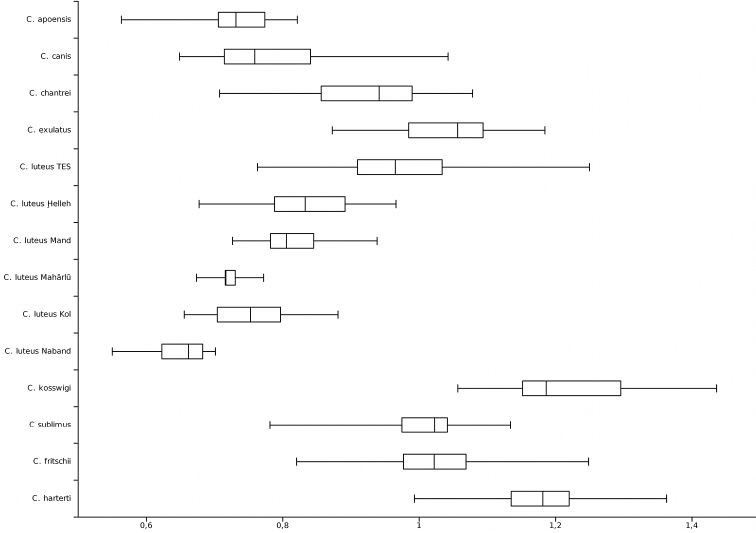
Last unbranched dorsal-fin ray length / head length; TES = Tigris-Euphrates system.

The dorsal fin and its base are rather short. It usually has four unbranched and 10 branched rays (Table 3). The last unbranched ray is considerably shorter than the head (Fig. 4), weakly ossified, and its distal part is flexible. The anal fin has three unbranched and six branched rays (Table 4). Pectoral and ventral fins are relatively short (Table 1).

**Table 3.** Number of branched dorsal-fin rays.

	**n**	**7**	**8**	**9**	**10**	**11**
*Carasobarbus apoensis*	66			2	63	1
*Carasobarbus canis*	90			5	85	
*Carasobarbus chantrei*	196			21	164	11
*Carasobarbus exulatus*	110		8	99	3	
*Carasobarbus fritschii*	297	1	23	268	5	
*Carasobarbus harterti*	30			30		
*Carasobarbus kosswigi*	23			3	20	
*Carasobarbus luteus*	441		1	23	411	6
Naband population	10			1	9	
*Carasobarbus sublimus*	18			2	16	

**Table 4.** Number of branched anal-fin rays.

	**n**	**5**	**6**	**7**
*Carasobarbus apoensis*	65		65	
*Carasobarbus canis*	90	2	88	
*Carasobarbus chantrei*	197	3	194	
*Carasobarbus exulatus*	109	3	106	
*Carasobarbus fritschii*	296	3	293	
*Carasobarbus harterti*	30		29	1
*Carasobarbus kosswigi*	23		23	
*Carasobarbus luteus*	439	3	435	1
Naband population	10		10	
*Carasobarbus sublimus*	18		18	

**Figure 5.**
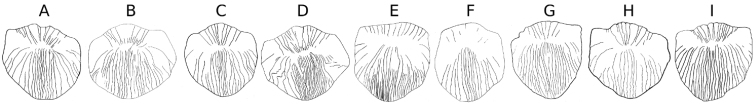
Striation pattern of scales taken from anteriour part of the boby above lateral line. **A** *Carasobarbus apoensis* **B** *Carasobarbus canis* **C** *Carasobarbus chantrei* **D** *Carasobarbus exulatus* **E** *Carasobarbus fritschii* **F** *Carasobarbus harterti* **G** *Carasobarbus kosswigi* **H** *Carasobarbus luteus* **I** *Carasobarbus sublimus*.

*Carasobarbus apoensis* has 27 to 32 scales in the lateral line (Table 5), usually 4.5 scales above the lateral line (Table 6), 3.5 or 4.5 scales below the lateral line (Table 7) and 12 scales around the least circumference of the caudal peduncle (Table 8). The scales are shown in Fig. 5.

**Table 5.** Lateral line scale count.

	**n**	**25**	**26**	**27**	**28**	**29**	**30**	**31**	**32**	**33**	**34**	**35**	**36**	**37**	**38**	**39**
*Carasobarbus apoensis*	60			1	9	15	20	14	1							
*Carasobarbus canis*	74					1	3	16	19	12	13	10				
*Carasobarbus chantrei*	168							5	11	31	48	36	29	7	1	
*Carasobarbus exulatus*	79		1	3	17	18	24	13	3							
*Carasobarbus fritschii*	264						1	12	21	39	75	58	36	15	4	3
*Carasobarbus harterti*	24							1		5	9	4	4		1	
*Carasobarbus kosswigi*	19								1	7	2	3	5		1	
*Carasobarbus luteus*	390	11	52	79	120	84	29	9	5	1						
Naband population	8				1	3	3	1								
*Carasobarbus sublimus*	11			4	3	4										

**Table 6.** Number of scales above the lateral line.

	**n**	**3,5**	**4**	**4,5**	**5**	**5,5**	**6**	**6,5**	**7**
*Carasobarbus apoensis*	60		2	45	7	6			
*Carasobarbus canis*	82			48	11	20	3		
*Carasobarbus chantrei*	171			4	1	147	6	13	
*Carasobarbus exulatus*	79		3	70	5	1			
*Carasobarbus fritschii*	276			15		226		35	
*Carasobarbus harterti*	28					4		24	
*Carasobarbus kosswigi*	21					8	5	7	1
*Carasobarbus luteus*	389	6	2	315	19	46	1		
Naband population	8			8					
*Carasobarbus sublimus*	17			16		1			

**Table 7.** Number of scales below the lateral line.

	**n**	**3**	**3,5**	**4**	**4,5**	**5**	**5,5**	**6**	**6,5**
*Carasobarbus apoensis*	57		14		41		2		
*Carasobarbus canis*	80		2	3	65	1	9		
*Carasobarbus chantrei*	173			1	84	3	84	1	
*Carasobarbus exulatus*	79		24	1	51	3			
*Carasobarbus fritschii*	286		7	3	151	5	117	1	2
*Carasobarbus harterti*	29		1		10		18		
*Carasobarbus kosswigi*	23				4	3	15		1
*Carasobarbus luteus*	384	2	125	16	231	9	1		
Naband population	8				8				
*Carasobarbus sublimus*	17		1		13	1	2		

**Table 8.** Number of scales around the least circumference of the caudal peduncle.

	**n**	**10**	**11**	**12**	**13**	**14**	**15**	**16**	**17**	**18**	**19**	**20**
*Carasobarbus apoensis*	60			58	2							
*Carasobarbus canis*	85			80	1	4						
*Carasobarbus chantrei*	168			4	7	110	27	20				
*Carasobarbus exulatus*	87	1	6	80								
*Carasobarbus fritschii*	253					3	12	212	26	23		1
*Carasobarbus harterti*	28				2	4	3	18	1			
*Carasobarbus kosswigi*	21			1	2	10	3	5				
*Carasobarbus luteus*	408	3	2	399	4							
Naband population	9			8	1							
*Carasobarbus sublimus*	17			17								

**Figure 6.**
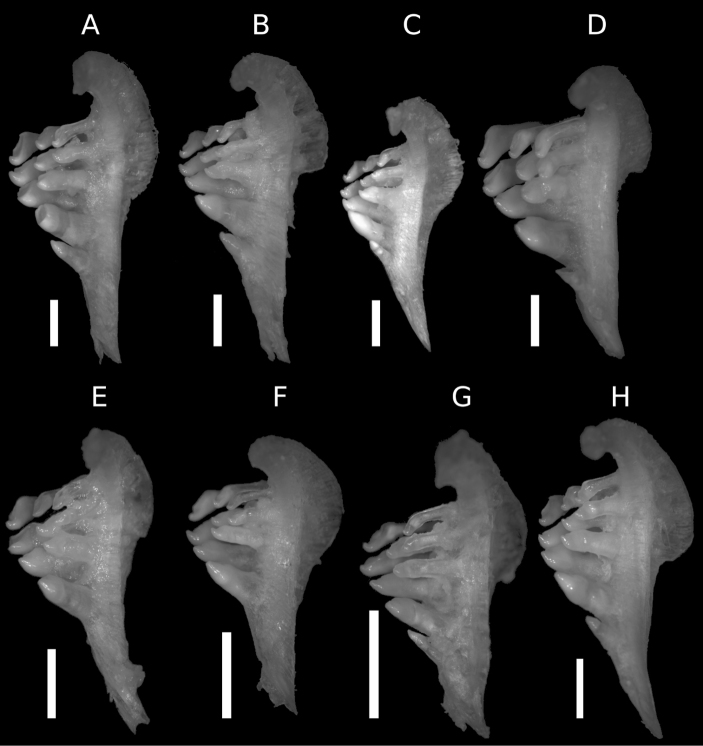
Pharyngeal bone. **A** *Carasobarbus apoensis* (SMF 30168, 190.1 mm SL) **B** *Carasobarbus canis* (SMF 30175, 168.7 mm SL) **C** *Carasobarbus chantrei* (SMF 33133, 165.9 mm SL) **D** *Carasobarbus exulatus* (SMF 33107, 170.1 mm SL) **E** *Carasobarbus fritschii* (SMF 33405, 147.2 mm SL) **F** *Carasobarbus harterti* (SMF 33396, 105.9 mm SL) **G** *Carasobarbus kosswigi* (SMF 30174, 141.5 mm SL) **H** *Carasobarbus luteus* (SMF 30179, 143.4 mm SL). Scale bar = 3 mm.

The pharyngeal teeth count is 2.3.5- in 12 specimens, -5.3.2 in two specimens and 1.3.5- in one specimen. The pharyngeal teeth are hooked at their tips (Fig. 6).

Live colouration is golden with olive fins. The upper side is darker than the belly (Fig. 2). In ethanol-preserved specimens the upper side is dark, the belly yellow and the fins are grey or yellow (Fig. 1). Juveniles have a dark lateral spot on the caudal peduncle.

The maximum length observed in the material examined is 288 mm SL.

*Carasobarbus apoensis* differs from all congeners, except *Carasobarbus luteus*, by having one rather than two pairs of barbels. For a comparison with *Carasobarbus luteus* populations see below.

**Figure 7.**
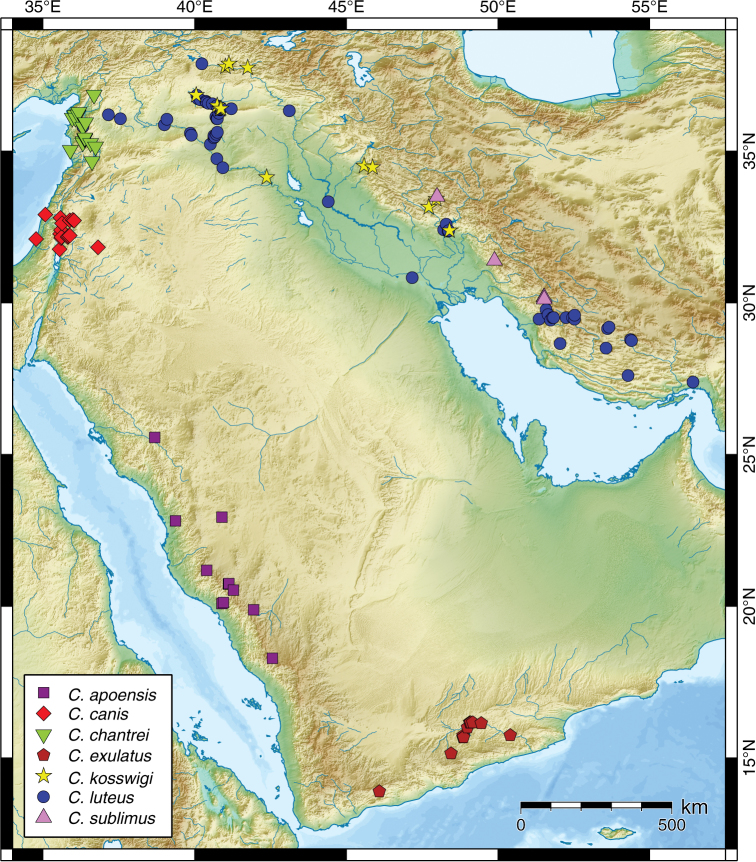
Map of the distribution of *Carasobarbus apoensis*, *Carasobarbus canis*, *Carasobarbus chantrei*, *Carasobarbus exulatus*, *Carasobarbus kosswigi*, *Carasobarbus luteus*, and *Carasobarbus sublimus*.

**Distribution.** *Carasobarbus apoensis* occurs in the Al Ḩijāz mountain range in wadis draining either inland or towards the Red Sea (Fig. 7). It is endemic to Saudi Arabia.

#### Habitats and biology.

This species inhabits the upper courses of wadis, which are characterised by strong seasonal fluctuations in water levels, temperature and other physiochemical parameters.

#### Conservation status.

*Carasobarbus apoensis* is rated Least Concern and still occurs in large numbers, but abstraction of large specimens by recreational fishing, water abstraction and habitat loss might become problematic for this species (BCEAW 2002).

#### Remarks and discussion.

*Carasobarbus apoensis* was originally described from Khamīs Mushayt, Wādī Turabah and Wādī Adamah as a member of the genus *Barbus* (Banister and Clarke 1977). It was later transferred to the genus *Carasobarbus* (Ekmek-çi and Banarescu 1998). Alkahem and Behnke (1983) reported an unknown *Barbus* and tentatively considered these specimens to be atypical *Carasobarbus apoensis*. We did not find any evidence of an undescribed *Carasobarbus* species that occurs sympatrically with *Carasobarbus apoensis*, thus we agree with their conclusion.

*Carasobarbus apoensis* is very closely related to *Carasobarbus luteus* (KB unpublished data).

### Carasobarbus canis

(Valenciennes in Cuvier and Valenciennes 1842)

http://species-id.net/wiki/Carasobarbus_canis

Barbus canis Valenciennes in Cuvier and Valenciennes 1842: 186.
Barbus beddomii Günther 1868: 110.

#### Material.

**Type material.** Lectotype of *Barbus canis*: MNHN 0000-1413, 1, Jordan River [31°46'N, 35°33'E], Bové, 1833 (designated by Krupp and Schneider 1989).

Paralectotype of *Barbus canis*: MNHN 0000-3944, 1, same data as lectotype.

Holotype of *Barbus beddomii*: BMNH 1863.11.3:5, 1, Lake Tiberias [32°48'N, 35°35'E], T. W. Beddome.

**Non-type material.** Jordan River Drainage. SMF 14075, 2, Lake Tiberias (32°48'N, 35°35'E), M. Goren, 15 Mar 1968. - SMF 33134, 16, Syria, Nahr al Yarmūk near Jallayn (32°44'21"N, 35°58'56"E), N. Alwan et al., 16 Oct 2008. - SMF 24464, 1, Jordan, Nahr al Yarmūk near Maqārin (32°43'N, 35°53'E), F. Krupp and W. Schneider, 23 Sep 1985. - SMF 30175, 11, Syria, Lake Muzayrīb [32°42'40"N, 36°1'39"E], F. Krupp and W. Schneider, 12 Apr 1989. - SMF 33135, 17, Jordan, Wadi al-‘Arab near the dam (32°37'6"N, 35°37'46"E), N. Alwan et al., 25 Oct 2008. - SMF 17123, 16, Wādī al Yābis (32°24'N, 35°36'E), F. Krupp and W. Schneider, 23 Jul 1980. - ZMH H 2343, 3, Jordan, Wādī Kufrinjah (32°16'25"N, 35°33'42"E). - SMF 24344, 3; SMF 24345, 17, Jordan, Nahr az Zarqā’ (32°12'N, 35°50'E), F. Krupp and W. Schneider, 22 Jul 1980. - SMF 24339, 3, Jordan, Nahr az Zarqā’ (32°10'N, 35°37'E), F. Krupp and W. Schneider, 21 Jul 1980. - SMF 24340, 1, Jordan, Nahr az Zarqā’ near Sadd al Malik Talal (32°10'N, 35°49'E), F. Krupp and W. Schneider, 22 Jul 1980. - SMF 24331, 7; SMF 24346, 3, Jordan, Nahr al Yarmūk channel (32°08'N, 35°36'E), F. Krupp and W. Schneider, 21 Jul 1980. - NMW 53961, 1, Jordan River [31°46'N, 35°33'E], Cenoni, Dec 1898.

Azraq Oasis. BMNH 1956.2.24:15-16, 2; BMNH 1965.11.24:2, 1, Jordan, wetland near Azraq ash Shīshān [31°50'N, 36°49'E].

Coastal rivers of the Mediterranean Sea. BMNH 1949.9.16:124, 1, Israel, Naẖal Na‘aman [32°54'42"N, 35°4'50"E]. - NMW 22367, 1, Israel, Naẖal Na‘aman [32°54'42"N, 35°4'50"E], H. Steinitz, 21 Oct 1955. - SMF 9229, 1, Israel, Naẖal Yarqon [32°6'7"N, 34°46'32"E].

#### Diagnosis.

Two pairs of barbels, 29 to 35 scales in the lateral line and usually 12 scales around the least circumference of the caudal peduncle, last unbranched ray of dorsal fin shorter than head.

#### Description.

**Figure 8.**
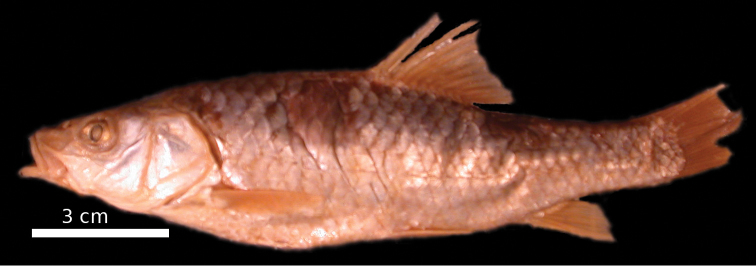
*Carasobarbus canis*, lectotype (MNHN 1413) from Jordan River.

**Figure 9.**
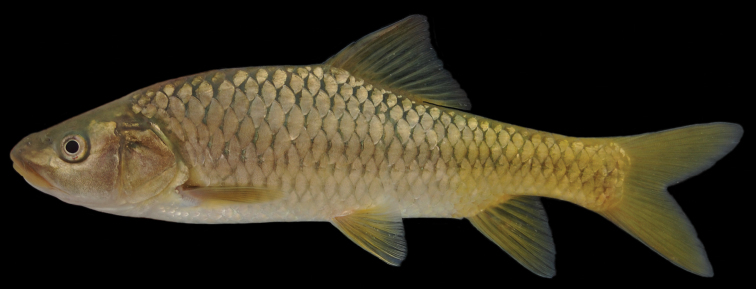
*Carasobarbus canis*, live specimen from Wadi al-‘Arab.

The body is low. A nuchal hump is present in adults but absent in juveniles. The largest body depth is at the origin of the dorsal fin. The head is long, rather low and fairly narrow with straight dorsal and convex ventral profile (Figs 8, 9). The head length approximately equals the body depth. The mouth is terminal or slightly subterminal. Two pairs of barbels are present (Table 2). The lips are smooth and thin (Fig. 3). The eyes are at the end of the anterior half of the head. The morphometric characters are summarised in Table 1.

Pectoral, ventral, dorsal and anal fins are comparatively short (Table 1). The dorsal fin usually has four unbranched and 10 branched rays (Table 3). The last unbranched ray is ossified and its distal part is flexible. It is usually markedly shorter than the head (Fig. 4). The anal fin usually has three unbranched and six branched fin rays (Table 4).

There are 29 to 35 scales in the lateral line (Table 5), usually 4.5 or 5.5 scales above the lateral line (Table 6), usually 4.5 scales below the lateral line (Table 7) and usually 12 scales around the least circumference of the caudal peduncle (Table 8). The scales are shown in Fig. 5.

The pharyngeal teeth count is 2.3.5-5.3.2 in 23 specimens, 2.3.3-5.3.2 in one specimen, 2.3.5- in one specimen and -5.3.2 in one specimen. The pharyngeal teeth are hooked at their tips (Fig. 6).

Live specimens are silvery to bronze coloured. The posterior third of the body and the fins are distinctly yellow in many specimens (Fig. 9). Ethanol-preserved specimens are brownish yellow and the back is only slightly darker than the rest of the body (Fig. 8). The fins are brownish yellow. Juveniles have a dark lateral spot on the caudal peduncle.

*Carasobarbus canis* differs from *Carasobarbus apoensis* and *Carasobarbus luteus* in having two pairs of barbels vs. one, from *Carasobarbus kosswigi* and *Carasobarbus sublimus* in having a crescent-shaped lower lip without median lobe vs. a spatulate lower lip with median lobe, from *Carasobarbus exulatus* in modally having 10 branched dorsal-fin rays vs. nine and from *Carasobarbus chantrei*, *Carasobarbus fritschii* and *Carasobarbus harterti* in modally having 10 scales around the least circumference of the caudal peduncle vs. 14 or 16.

#### Distribution.

*Carasobarbus canis* occurs in the Jordan River system (Fig. 7). There are only few records from coastal rivers of the Mediterranean Sea (Naẖal Na‘aman and Naẖal Yarqon). A recent treatment of the inland water fish communities of Israel does not report *Carasobarbus canis* from coastal rivers (Goren and Ortal 1999). The population in the Azraq Oasis was introduced by humans (Krupp and Schneider 1989). Since the year 2000 this species was not found in Azraq (Hamidan 2004) and the population may have disappeared due to drought. Records from the Tigris-Euphrates system (Banister 1980) are based on misidentifications.

#### Habitats and biology.

*Carasobarbus canis* inhabits a wide range of rivers, lakes and ponds (Goren 1974) with clean as well as polluted water (Mir 1990). Adults reach a length of about 40 cm (max. 66 cm) and are of economic importance locally (annual catch in Israel 1970-85 about 50 t, Fishelson et al. 1996). It feeds on fish, aquatic invertebrates, algae and detritus (Ben-Tuvia 1978, Spataru and Gophen 1985, Krupp and Schneider 1989). The relative proportion of fish in the diet increases with body length and small cyprinids of the genus *Mirogrex* are their most important prey (Spataru and Gophen 1985). The spawning grounds are (among others) at the shore of Lake Tiberias where the spawning occurs in shallow water over hard bottom in December and January, one month after the start of the rainy season (Fishelson et al. 1996). The sticky eggs attach to the substrate. Winter spawning is seen as evidence for an origin in cooler areas (Fishelson et al. 1996).

#### Conservation status.

Catches in Lake Tiberias are declining (Fishelson et al. 1996). The species is rated Least Concern by the IUCN (Crivelli 2006a). The population in Lake Tiberias does not face serious threats; the riverine populations are declining and threatened by pollution, water extraction, drought and fragmentation due to damming (Crivelli 2006a).

#### Remarks and discussion.

*Carasobarbus canis* was described from the Jordan River as a member of the genus *Barbus* (Cuvier and Valenciennes 1842). Later it was assigned to *Luciobarbus* (Heckel 1843), and *Labeobarbus* (Günther 1864). Subsequently it was transfered back to *Barbus* (Günther 1868) and then placed in *Tor* (Karaman 1971, Banarescu 1977), *Barbus* (Banister and Clarke 1977, Krupp 1983a), *Carasobarbus* (Ekmekçi and Banarescu 1998) and *Barbus* subgenus *Carasobarbus* (Tsigenopoulos et al. 2010). MNHN 0000-1413 was designated as lectotype (Krupp and Schneider 1989). *Barbus beddomii* is considered to be a junior synonym of *Carasobarbus canis* (Berg 1949, Karaman 1971, Krupp and Schneider 1989).

### Carasobarbus chantrei

(Sauvage, 1882)

http://species-id.net/wiki/Carasobarbus_chantrei

Labeobarbus chantrei Sauvage 1882: 165.
Barynotus verhoeffi Battalgil 1942: 292.

#### Material.

**Type material.** Lectotype of *Labeobarbus chantrei*: MNHN A-3866, Turkey, Amik Gölü [36°12'24"N, 36°9'26"E], H. Chantre, 1881 (designated by Krupp 1985a).

Paralectotypes of *Labeobarbus chantrei*: MNHN A-3937, 1, same data as lectotype. - MNHN A-3938, 2; MNHN A-3939, 3; MNHN A-3940, 1, Syria, Ḩamāh [35°9'0"N, 36°43'59"E], H. Chantre, 1881.

**Non-type material.** Orontes River drainage. MNHN B-2977, 1, Syria, Orontes, A. Gruvel, 1829. - BMNH 1934.1.25:4, 1, Syria, Orontes. - FSJF 2311, 11, Turkey, Karasu Çayı below dam of Tahtaköprü Barajı (36°51'7"N, 36°41'10"E), M. Özulug and J. Freyhof, 7 Nov 2007. - SMF 17115, 8, Turkey, Orontes, 8 km E of Hatay (36°17'N, 36°11'E), J. Winkler and B. Koster, 20 Sep 1982. - CMNFI 88-0019, 1, Turkey, 8 km southwest of Hatay (36°11'N, 36°3'E). - SMF 17110, 4, Turkey, tributary to Orontes (36°11'N, 36°3'E), F. Krupp, 23 Aug 1978. - SMF 17122, 2, Turkey, 2 km southeast of Samandağı (36°6'N, 35°58'E), F. Krupp, 23 Aug 1978. - FSJF uncatalogued, 16, Turkey, at Sinanlı (36°5'51"N, 36°4'43"E), M. Özulug and J. Freyhof, 8 Nov 2007. - SMF 33130, 40, Syria, near Mashra’a el Būz (35°57'3"N, 36°23'45"E), N. Alwan et al., 8 Oct 2008. - SMF 33131, 58, Syria, ‘Ayn az Zarqa (35°56'40"N, 36°24'9"E), N. Alwan et al., 8 Oct 2008. - SMF 17107, 1, Syria, Jisr ash Shughūr (35°48'N, 36°19'E), F. Krupp, 20 Aug 1980. - SMF 17109, 2, Syria, main bridge at Jisr ash Shughūr (35°48'N, 36°19'E), F. Krupp, 19 Aug 1978. - CMNFI 88-0018, 4, Syria, ‘Ayn Zaqa (35°27'N, 36°23'E). - SMF 17114, 1; SMF 17121, 7, Syria, ‘Ayn Zaqa (35°27'N, 36°23'E), F. Krupp, 25–27 Mar 1979. - BMNH 1968.12.13:188-190, 3, Syria, spring lake at Qal‘at al Maḑīq [35°25'N, 36°23'E]. - SMF 17120, 7, Syria, aquaculture pond near Qal‘at al Maḑīq (35°25'N, 36°23'E), F. Krupp, 8 Aug 1978. - SMF 33132, 5, Syria, stream at Qal‘at al Jarras (35°19'49"N, 36°18'38"E), N. Alwan et al., 12 Oct 2008. - SMF 17111, 6, Syria, ‘Ašārna (35°17'N, 36°19'E), F. Krupp, 11 Aug 1978. - SMF 17117, 5, Syria, near Shayzar (35°16'N, 36°34'E), F. Krupp, 27 Mar 1979. - SMF 24349, 3, Syria, Shayzar (35°16'N, 36°34'E), F. Krupp and W. Schneider, 17 Aug 1980. - SMF 17118, 1, Syria, 200 m below western outlet of Lake Homs (34°40'N, 36°37'E), F. Krupp and W. Schneider, 3 Aug 1978. - SMF 17119, 5, Syria, western outlet of Lake Homs (34°40'N, 36°37'E), F. Krupp and W. Schneider, 3 Aug 1978. - SMF 33133, 24, Syria, Lake Homs at Qaţţīnah (34°39'43"N, 36°37'6"E), N. Alwan et al., 13 Oct 2008.

Mediterranean coastal rivers. SMF 31669, 1; SMF 31670, 1, Syria, Nahr Marqīyah (35°1'50"N, 35°54'18"E), N. Alwan et al., 10 Oct 2008.

Tigris-Euphrates system. SMF 12966, 1, Turkey, Balıklıgöl at Şanlıurfa [37°8'52"N, 38°47'4"E], L. Lortet, 1884.

#### Diagnosis.

Two pairs of barbels, 31 to 38 scales in the lateral line and usually 14 to 16 scales around the least circumference of the caudal peduncle, last unbranched dorsal-fin ray equal to or shorter than head.

#### Description.

**Figure 10.**
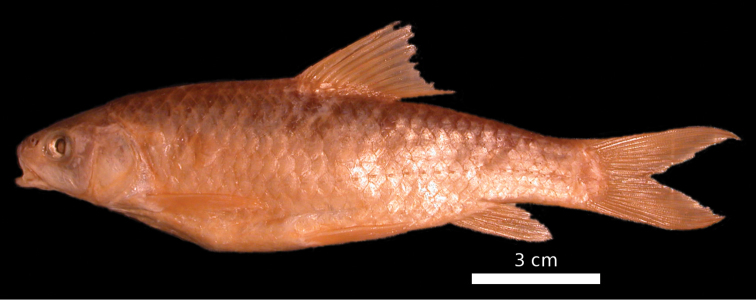
*Carasobarbus chantrei*, paralectotype (MNHN A-3939) from Orontes at Ḩamāh.

**Figure 11.**
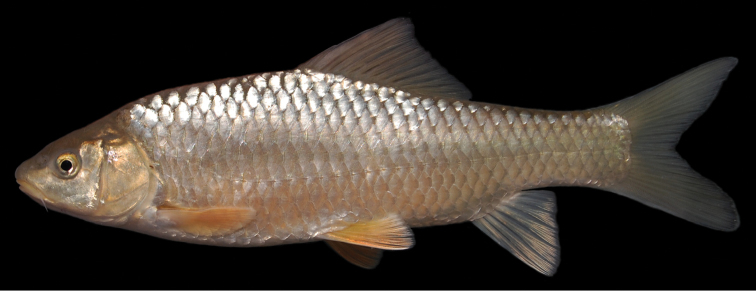
*Carasobarbus chantrei*, live specimen from Buḩayratt Qaţţīnah.

**Figure 12.**
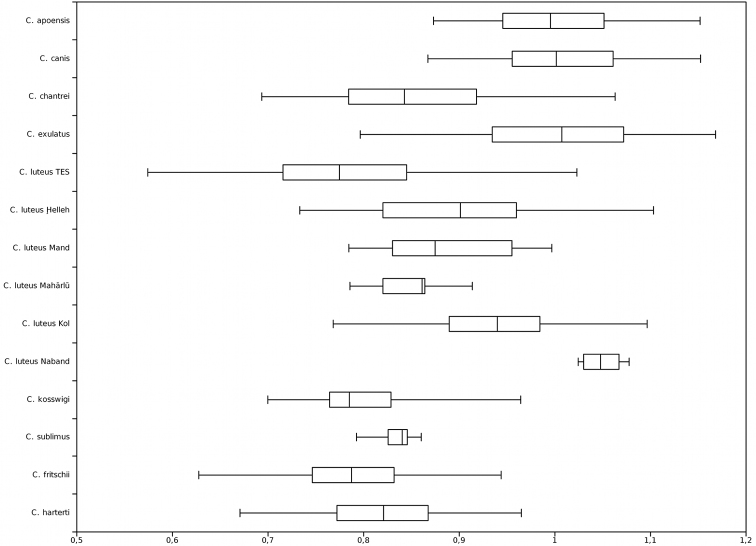
Head length / body depth; TES = Tigris-Euphrates system.

The body is comparatively high-backed and laterally compressed in mid-sized specimens but low-backed and almost cylindrical in large specimens. In large specimens a pronounced nuchal hump is present, in smaller specimens it is only weakly developed or absent. The maximum body depth is at the origin of the dorsal fin. The head is short and blunt with a convex ventral profile and a slightly convex to straight dorsal profile (Figs 10, 11). The mouth is terminal or slightly sub-terminal with two pairs of short barbels (Table 2).The body depth is usually greater than the head length (Fig. 12).The eyes are slightly protuberant and lie at the end of the anterior half of the head. The morphometric characters are summarised in Table 1.

The dorsal fin usually has four unbranched and nine to 11 branched rays (Table 3). The last unbranched ray is ossified but not very thick and flexible in its distal part. It is usually shorter than the head (Fig. 4). The anal fin usually has three unbranched and five or six branched rays (Table 4).

There are 31 to 38 scales in the lateral line (Table 5), 4.5 to 6.5 scales above the lateral line (Table 6), four to six scales below the lateral line (Table 7) and 12 to 16 scales around the least circumference of the caudal peduncle (Table 8). The scales are shown in Fig. 5.

The pharyngeal teeth count is 2.3.5-5.3.2 in two specimens, 2.3.5- in 11 specimens, -5.3.2 in two specimens and 1.3.5- in one specimen. The pharyngeal teeth are hooked at their tips (Fig. 6).

Small live specimens are silvery; larger specimens are silvery or bronze coloured and sometimes have yellow pectoral and ventral fins (Fig. 11). Small ethanol-preserved specimens are silvery with a somewhat darker back and a salmon pink hue. Juveniles have a dark lateral spot on the caudal peduncle. Ethanol-preserved adults are yellow-brown and the back is only slightly darker than the rest of the body (Fig. 10).

The maximum length observed in the material examined is 385 mm SL.

*Carasobarbus chantrei* differs from *Carasobarbus apoensis*, *Carasobarbus canis*, *Carasobarbus exulatus*, *Carasobarbus luteus* and *Carasobarbus sublimus* in having 31 to 38 scales in the lateral line vs. 27 to 32, 29 to 35, 26 to 32, 25 to 33 and 27 to 29 respectively and modally 14 scales around the least circumference of the caudal peduncle vs. 12. It differs from *Carasobarbus kosswigi* and *Carasobarbus sublimus* in having a crescent-shaped lower lip without median lobe vs. a spatulate lower lip with median lobe and from *Carasobarbus exulatus*, *Carasobarbus fritschii* and *Carasobarbus harterti* in modally having 10 branched dorsal-fin rays vs. nine.

#### Distribution.

*Carasobarbus chantrei* occurs in the Orontes river drainage system (Fig. 7). Two juvenile specimens where collected in Nahr Marqīyah, a coastal river in Syria. This species had never before been reported from this location (Krupp 1985a) and it has most likely been introduced by humans. Two potential records from Nahr Quwayq (MNHN A-3861, MGHN 3554) are discussed in Krupp (1985a, c). Locality data for MHNL 3554 are ambiguous (Krupp 1985a). The locality for MNHN A-3861 is given as “Syria, Aleppo” in Krupp (1985a) and considered to be from Nahr Quwayq. The collection database of the MNHN gives “Origine: Syrie, localité: Alep, Milieu: Continent, Bassin hydrologique: Asi, Cours d’eau: Asi” as locality. As these data are contradictory, it is likely that the specimens do not come from the Nahr Quwayq, but from the Orontes (=Asi) and *Carasobarbus chantrei* does probably not occur in the Nahr Quwayq. A record from the Ceyhan Nehri (Krupp 1985c) is not backed by specimens. Records from the Tigris-Euphrates basin are misidentified *Carasobarbus luteus* (Krupp 1985a, Krupp and Schneider 1991) and the specimen from Balıklıgöl at Şanlıurfa in Turkey (SMF 12966) is probably mislabelled or was introduced there (Krupp 1985a). It is not included in the map.

#### Habitats and biology.

*Carasobarbus chantrei* occurs in a wide range of habitats stretching from stagnant waters of lakes to rapidly flowing river courses.

#### Conservation status.

*Carasobarbus chantrei* is utilised as food fish locally but is increasingly replaced by carp (Krupp 1985a). During a field survey in Syria in 2008, the species was still abundant in parts of the Orontes. However, large stretches of this river, especially in the Al Ghāb area, suffer heavily from water abstraction and pollution by sewage and domestic waste and are devoid of fish. The species is rated “Endangered B1ab(ii,iii)” by the IUCN (Crivelli 2006b). The main threat is habitat degradation due to water extraction, pollution and drought (Crivelli 2006b).

#### Remarks and discussion.

*Carasobarbus chantrei* was described from the Orontes and placed in *Labeobarbus* by Sauvage (1882). He transferred it to *Barbus* two years later (Sauvage 1884). In 1942 *Barynotus verhoeffi* was described from Amik Gölü, Turkey (Battalgil 1942). Ladiges (1960) erroneously synonymised *Barynotus verhoeffi* with *Carasobarbus canis*. Karaman (1971) synonymised *Carasobarbus chantrei* with *Carasobarbus canis* and thus transferred it to the genus *Tor* (sensu Karaman 1971). Fowler (1976) transferred *Barynotus verhoeffi* to the genus *Barbellion*. In 1985 Krupp redescribed *Carasobarbus chantrei* as a valid species and provisionally placed it into the genus *Barbus* sensu lato. He found the type series to be inhomogeneous (MNHN B-2889 are ‘*Barbus*’ *grypus*) and designated MNHN A-3866 as lectotype of *Carasobarbus chantrei* (Krupp 1985a). The ‘Catalog of Fishes’ does not list MNHN B-2889 as types for *Carasobarbus chantrei* (Eschmeyer 2011). Ekmekçi and Banarescu (1998) transferred the species to the genus *Carasobarbus*. Tsigenopoulos et al. (2010) used *Barbus* subgenus *Carasobarbus*.

### Carasobarbus exulatus

(Banister & Clarke, 1977)

http://species-id.net/wiki/Carasobarbus_exulatus

Barbus exulatus Banister and Clarke 1977: 116.

#### Material.

**Type material.** Holotype of *Barbus exulatus*: BMNH 1976.4.7:299, Yemen, Wādī Ḩaḑramawt at Qasam (16°10'N, 49°4'E), W. A. King-Webster.

Paratypes of *Barbus exulatus*: BMNH 1976.4.7:308, 1; BMNH 1976.4.7:300-307, 8, same data as holotype. - BMNH 1976.4.7:328-329, 2; BMNH 1976.4.7:330-331, 2, Yemen, Wādī ‘Idim/Wādī Ḩaḑramawt at Ghuraf (16°0'N, 49°0'E), W. A. King-Webster. - BMNH 1976.4.7:309, 1; BMNH 1976.4.7:310-318, 9; BMNH 1976.4.7:319-327, 9, Yemen, Wādī Ḩaḑramawt at Ghayl ‘Umar (15°44'N, 48°51'E), W. A. King-Webster. - BMNH 1976.4.7:332-333 probably Wādī Marrān in Wādī Aḩwar system [13°53'51"N, 46°05'14"E], G. Popov, 2 Aug 1962.

**Non-type material.** Wādī Ḩaḑramawt/al Masīlah drainage. BMNH 1976.5.17:9-10, 2, Yemen, Wādī al Khūn (16°10'N, 49°10'E). - SMF 33108, 10, Yemen, Wādī al Khūn (16°9'51"N, 49°6'2"E), F. Krupp et al., 3 Jun 2005. - SMF 33109, 17, Yemen, Wādī al Khūn (16°9'45"N, 49°4'46"E), F. Krupp et al., 3 Jun 2005. - SMF 33110, 14, Yemen, Wādī al Masīlah near Fughmah (16°8'36"N, 49°27'7"E), F. Krupp et al., 4 Jun 2005. - SMF 33111, 1, Yemen, Wādī al Masīlah at al Hind (15°44'53"N, 50°24'32"E), F. Krupp et al., 5 Jun 2005. - SMF 33106, 8, Yemen, Wādī ‘Idim at Ghayl ‘Umar near Arḑ ar Raydah (15°40'51"N, 48°51'59"E), F. Krupp et al., 2 Jun 2005. - SMF 33107, 11, Yemen, Wādī ‘Idim near Ghayl ‘Umar (15°40'10"N, 48°51'4"E), F. Krupp et al., 2 Jun 2005. -SMF 33105, 13, Yemen, Wādī Mara in Wādī Daw‘an system (15°8'36"N, 48°26'58"E), F. Krupp et al., 31 May 2005.

#### Diagnosis.

Dorsal fin with 9 branched rays in most specimens; last unbranched ray of dorsal fin as long as or longer than head; 2 pairs of barbels; 26 to 32 scales in the lateral line and usually 12 scales around the least circumference of the caudal peduncle.

#### Description.

**Figure 13.**
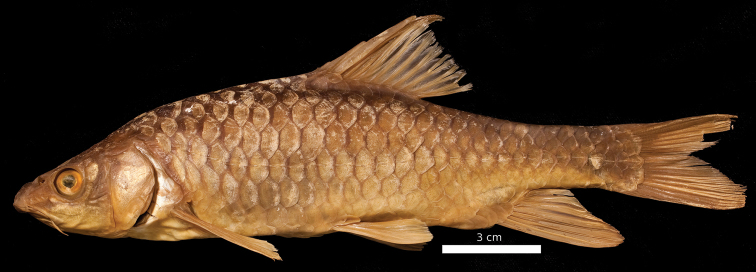
*Carasobarbus exulatus*, holotype (BMNH 1976.4.7:299) from Wādī Ḩaḑramawt at Qasam, ^©^ The Natural History Museum, London, photo P. Hurst.

**Figure 14.**
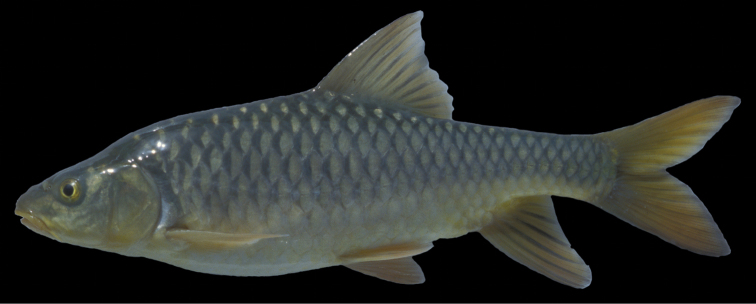
Adult *Carasobarbus exulatus*, live specimen from Wādī al Khūn.

**Figure 15.**
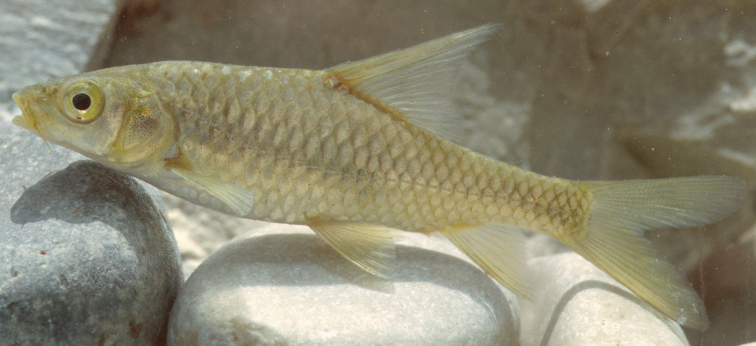
Juvenile *Carasobarbus exulatus*, live specimen from Wādī al Khūn.

The body is not particularly high backed and the maximum body depth is at the origin of the dorsal fin or slightly in front of it (Fig. 13). A nuchal hump is present in adult specimens (Fig. 14) but absent in juveniles (Fig. 15). The caudal peduncle is slender. The head profile is convex ventrally and straight dorsally. The body depth is about the same as the head length (Fig. 12). In specimens below 100 mm SL, the head is rather narrow, in larger specimens it becomes wider. The mouth is subterminal and comparatively narrow. Two pairs of barbels are present (Table 2), the posterior one is rather long. The eyes are at the end of the anterior half of the head and slightly protuberant. The morphometric characters are summarised in Table 1.

The dorsal fin is long and usually has four unbranched and eight to 10 branched rays (Table 3). The last unbranched ray is strongly ossified and only the tip is flexible. Its length is about the same as the head length (Fig. 4). The anal fin is long, usually has three unbranched and five or six branched rays (Table 4).

There are 26 to 32 scales in the lateral line (Table 5), 4 to 5.5 scales above the lateral line (Table 6), 3.5 to five scales below the lateral line (Table 7) and 10 to 12 scales around the least circumference of the caudal peduncle (Table 8). The scales are shown in Fig. 5.

The pharyngeal teeth count is 2.3.5-5.3.2 in one specimen, 2.3.5- in 16 specimens, -5.3.2 in one specimen and 2.3.4- in one specimen. The pharyngeal teeth are hooked at their tips (Fig. 6).

In live specimens and freshly preserved specimens the back and the sides are grey to golden, the belly is yellowish white and the fins are sometimes golden to orange (Fig. 15). Preserved specimens have a dark back and a lighter belly, the fins are whitish or greyish. Juveniles have a dark spot on the sides of the caudal peduncle.

The maximum length observed in the material available is 288 mm SL.

*Carasobarbus exulatus* differs from all congeners, except *Carasobarbus fritschii* and *Carasobarbus harterti* in modally having nine instead of 10 branched dorsal-fin rays. It differs from *Carasobarbus fritschii* and *Carasobarbus harterti* in modally having 12 scales around the least circumference of the caudal peduncle vs. 16 and in having 26 to 32 scales the lateral line vs. 30 to 39 and 31 to 38 respectively.

#### Distribution.

This species is endemic to Yemen and occurs in Wādī Ḩaḑramawt / Wādī al Masīlah and its pleistocene tributaries (Banister and Clarke 1977, Krupp 1983a, Fig. 7). It is also known from Sadd Ma’rib (Al-Safadi 1995), a dam lake at 15°23'46"N, 45°14'37"E and Wādī Ḩajr (14°02'42"N, 48°40'27"E), where they are “found throughout the whole year and are distributed all over the stream” (Attaala and Rubaia 2005).

Locality data for BMNH 1976.4.7:332-333 is given as “Wadi Maran, E. Yemen” (Banister and Clarke 1977), which is most likely Wādī Marrān [13°53'51"N, 46°05'14"E], representing the westernmost record of this species that is backed by specimens.

#### Habitats and biology.

The biology of this species is mostly unknown.

#### Conservation status.

During a field expedition in 2005 one of the authors saw large, continuous water bodies in the Wādī Ḩaḑramawt / Wādī al Masīlah area. The species is rated as “Endangered B1a, b; B2a, b” and water extraction is identified as the main threat (BCEAW 2002).

#### Discussion.

*Carasobarbus exulatus* was described from Wādī Ḩaḑramawt and Wādī Maran in Yemen and placed in *Barbus* (Banister and Clarke 1977). Later it was transferred to *Carasobarbus* (Ekmekçi and Banarescu 1998).

### Carasobarbus fritschii

(Günther, 1874) comb. n.

http://species-id.net/wiki/Carasobarbus_fritschii

Barbus fritschii Günther 1874: 231.
Barbus rothschildi Günther 1901: 368.
Barbus riggenbachi Günther 1902: 447.
Capoeta atlantica Boulenger 1902: 124.
Capoeta waldoi Boulenger 1902: 124.
Barbus paytonii Boulenger 1911: 82.

#### Material.

**Type material.** Syntypes of *Barbus fritschii*: BMNH 1874.1.30:27-31, 5, Morocco, Oued Ksob in Oued Igrounzar drainage [31°28'59"N, 9°46'3"W], K. v. Fritsch and J. Rein, 1872.

Syntypes of *Barbus paytonii*: BMNH 1903.10.29:17-20, 7, Morocco, Oued Oum er Rbia [33°19'40"N, 8°20'2"W], F. W. Riggenbach.

Syntypes *Barbus riggenbachi*: BMNH 1902.7.28:20-21, 2, Morocco, Oued Oum er Rbia [33°19'40"N, 8°20'2"W], F. W. Riggenbach. - BMNH 1902.7.28:19, 1, Morocco, Oued Talmest [31°52'15"N, 9°18'31"W], F. W. Riggenbach.

Syntypes *Barbus rothschildi*: BMNH 1901.7.26:6-7, 2, Morocco, Oued Oum er Rbia [33°19'40"N, 8°20'2"W], E. Hartert.

Syntypes of *Capoeta atlantica*: BMNH 1902.1.4:18-19, 2, Morocco, Oued Nfis at Trigadir-el-hor (Tagadirt n’Bour?) [31°9'21"N, 8°6'2"W], E. G. B. Meade-Waldo.

Syntypes of *Capoeta waldoi*: BMNH 1902.1.4:16-17, 2, Morocco, Oued Nfis at Trigadir-el-hor (Tagadirt n’Bour?) [31°9'21"N, 8°6'2"W], E. G. B. Meade-Waldo.

**Non-type material.** Oued al Maleh drainage. SMF 33412, 5; SMF 33510, 1; SMF 33511, 1; SMF 33512, 1, Morocco, Oued al Maleh above the dam (33°33'53"N, 7°22'3"W), K. Borkenhagen and J. Freyhof, 19 Apr 2011. - MNHN 1919-0365, 1; MNHN 1919-0366, 1, Morocco, Oued Bou Asseïla near Chaouia [33°19'34"N, 7°16'46"W], H. Millet, 1919.

Oued Bou Regreg drainage. MNHN 1939-0124, 1, Morocco, Oued Akrech [33°56'7"N, 6°47'41"W], J. M. Pérès, 1939. - SMF 33411, 10; SMF 33503, 1; SMF 33504, 1; SMF 33505, 1, Morocco, Oued Korifla above the dam lake (33°44'0"N, 6°43'43"W), K. Borkenhagen and J. Freyhof, 18 Apr 2011.

Oued Igrounzar drainage. BMNH 1889.7.19:9, 1, Morocco, near Essaouira [31°30'45"N, 9°46'12"W], C. Payton. - SMF 636, 4; SMF 952, 6, Morocco, Oued Ksob [31°28'59"N, 9°46'3"W], K. v. Fritsch and J. Rein, 1872. - SMF 33405, 19; SMF 33446, 1; SMF 33450, 1; SMF 33451, 1, Morocco, Oued Ksob near Essaouira (31°28'0"N, 9°45'32"W), A. Azeroual et al., 11 Apr 2011. - SMF 33388, 1; SMF 33389, 1; SMF 33390, 1; SMF 33404, 20, Oued Igrounzar between Ounara and El Ghazouane (31°27'21"N, 9°41'4"W), A. Azeroual et al., 10 Apr 2011. - SMF 33406, 2, Oued Igrounzar near El Khemis des Meskala (31°21'31"N, 9°24'20"W), A. Azeroual et al., 11 Apr 2011.

Oued Iqem drainage. SMF 33509, 1, Morocco, Oued Iqem near Skhirat (33°53'22"N, 6°59'56"W), K. Borkenhagen and J. Freyhof, 19 Apr 2011.

Oued Kiss drainge. MNHN 1924-0174, 1, Algeria, Oued Kiss at Marsa Ben Mehid (35°4'59"N, 2°10'1"W), C. A. Alluaud, 1924.

Oued Moulouya drainage. SMF 33407, 8; SMF 33408, 4; SMF 33479, 1; SMF 33481, 1; SMF 33484, 1, Morocco, Oued Za near Guefaït (34°13'36"N, 2°23'34"W), K. Borkenhagen and J. Freyhof, 15 Apr 2011. - MNHN 1926-0070, 1, Morocco, Oued Melloulou near Guercif [34°13'32"N, 3°21'13"W], P. M. Pallary, 1926. - NMW 19533, 1, Morocco, Ras el Aïn near Aïn Beni Mathar (=Berguent) [34°0'41"N, 2°1'47"W], F. Werner. - NMW 19532, 1, Morocco, Oued Za [32°57'0"N, 5°12'0"W], F. Werner.

Oued Oum er Rbia drainage. BMNH 1902.7.28:22-26, 5, Morocco, Oued Oum er Rbia [33°19'40"N, 8°20'2"W], F. Riggenbach. - BMNH 1903.7.1:8, 2, Morocco, El Jadida [33°15'18"N, 8°30'22"W], F. Riggenbach. - MNHN 1927-0099, 1; MNHN 1927-0100, 1; MNHN 1989-0535, 1, Morocco, Oued Oum er Rbia near Khenifra [32°56'21"N, 5°40'7"W], A. Gruvel and R. Dollfus, 1927. - MNHN 1928-0054, 1; MNHN 1928-0055, 1, Morocco, Oued Oum er Rbia near Khenifra [32°56'21"N, 5°40'7"W], P. Pallary, 1928. - SMF 33513, 1; SMF 33514, 1; SMF 33515, 1, Morocco, Oued Oum er Rbia near Boulaouane (32°51'33"N, 8°2'41"W), K. Borkenhagen and J. Freyhof, 20 Apr 2011. - SMF 33344, 1; SMF 33345, 1; SMF 33346, 1; SMF 33394, 12, Morocco, Oued Srou at bridge between Tighassaline and Khenifra (32°49'51"N, 5°36'36"W), A. Azeroual et al., 7 Apr 2011. - SMF 33360, 1; SMF 33361, 1; SMF 33362, 1; SMF 33395, 17, Morocco, Oued Derra near Oulad Yaïch (32°26'23"N, 6°19'24"W), A. Azeroual et al., 9 Apr 2011. - SMF 33363, 1; SMF 33364, 1; SMF 33365, 1; SMF 33397, 22, Morocco, Oued Oum er Rbia (32°18'53"N, 6°54'33"W), A. Azeroual et al., 9 Apr 2011.

Oued Sebou drainage. SMF 33410, 9; SMF 33494, 1; SMF 33495, 1; SMF 33496, 1, Morocco, Oued Ouergha between Sidi Qacem and Ouazzane (34°27'52"N, 5°30'39"W), K. Borkenhagen and J. Freyhof, 17 Apr 2011. - MNHN 1939-0125, 1; MNHN 1939-0126, 1; MNHN 1939-0127, 1, Morocco, El Gharb [34°25'N, 6°20'W], J. M. Pérès, 1939. - MNHN 1939-0122, 2; MNHN 1939-0123, 2; MNHN 1939-0145, 1, Morocco, Oued Sebou [34°15'53"N, 6°41'5"W], J. M. Pérès, 1939. - SMF 33409, 15; SMF 33489, 1; SMF 33491, 1; SMF 33493, 1, Morocco, Oued Lahdar near Taza (34°14'35"N, 4°3'55"W), K. Borkenhagen and J. Freyhof, 16 Apr 2011. - MNHN 1924-0191, 3, Morocco, Oued Beth near Dar Bel Hamri [34°11'14"N, 5°57'54"W], C. A. Alluaud, 1924. - MNHN 1920-0061, 1; MNHN 1920-0062, 1, Morocco, Oued Bou Hellou [34°9'19"N, 4°25'33"W], P. M. Pallary, 1920. - MNHN 1922-0065, 1, Morocco, Moulay Yacoub [34°5'17"N, 5°10'54"W], C. A. Alluaud, 1922. - MNHN 1920-0202, 1, Morocco, Faraoun near Volubilis [34°4'25"N, 5°33'25"W], C. A. Alluaud, 1920. - MNHN 1939-0128, 1; MNHN 1939-0129, 1, El Mabbabat [?], J. M. Pérès, 1939.

Oued Tennsift drainage. BMNH 1904.11.28:60, 1; BMNH 1905.11.28:60-63 and BMNH 1904.11.28:57-58, 6, Morocco, Oued Chichaoua [31°43'48"N, 8°49'48"W], F. Riggenbach. - MNHN 1919-0379, 1; MNHN 1919-0380, 1; MNHN 1919-0381, 1; MNHN 1919-0382, 1, Morocco, Oued Nfis near Dar Goundafi [31°43'41"N, 8°21'1"W], P. M. Pallary, 1919. - MNHN 1988-1146, 4, Morocco, Oued Nfis [31°43'41"N, 8°21'1"W], Goubier, VI.1988. - MNHN 1922-0066, 1; MNHN 1922-0067, 1; MNHN 1922-0068, 1, Morocco, Oued Chichaoua near Chichaoua [31°32'37"N, 8°45'46"W], C. A. Alluaud, 1922. - SMF 33371, 1; SMF 33372, 1; SMF 33373, 1; SMF 33374, 1; SMF 33398, 3, Morocco, Oued Nfis near Tameslouht (31°27'2"N, 8°8'22"W), A. Azeroual et al., 10 Apr 2011. - SMF 33378, 1; SMF 33379, 1; SMF 33380, 1; SMF 33399, 14; SMF 33403, 22, Morocco, Oued Nfis near Ouirgane (31°13'24"N, 8°6'50"W), A. Azeroual et al., 10 Apr 2011. - MNHN 1925-0371, 1, Morocco, Oued Nfis near Ouirgane [31°10'40"N, 8°4'24"W], J. Pellegrin, 1925.

#### Diagnosis.

Two pairs of barbels, 30 to 39 scales in the lateral line and 14 to 20 scales around the least circumference of the caudal peduncle; dorsal fin usually shorter than anal fin and more than 15 % of its last unbranched ray flexible, dorsal profile of the head convex.

#### Description.

**Figure 16.**
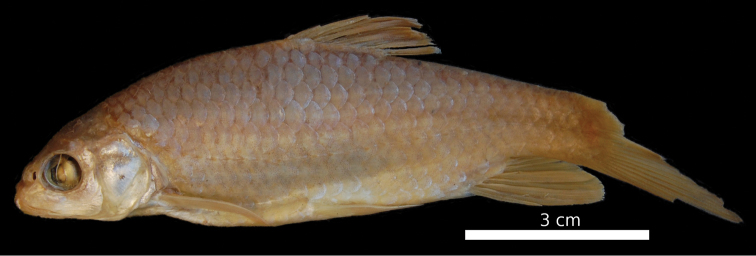
*Carasobarbus fritschii*, syntype (BMNH 1874.1.30:27-31) from Oued Ksob, ^©^ The Natural History Museum, London.

**Figure 17.**
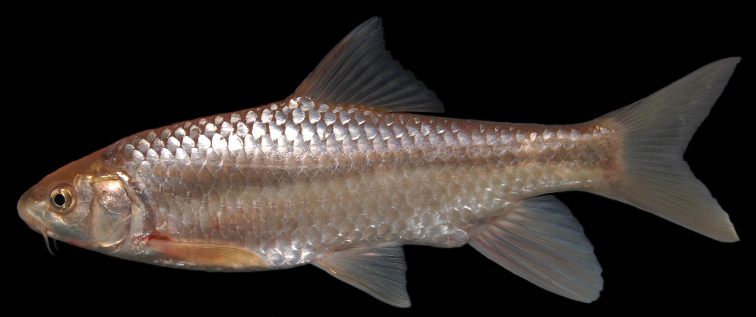
*Carasobarbus fritschii*, from Oued Ksob.

The body is of moderate height and sometimes has a small nuchal hump in larger specimens. The head is round with a convex dorsal profile and convex or straight ventral profile (Figs 16, 17). The head length is shorter than the body depth (Fig. 12), the mouth is inferior with two pairs of barbels (Table 2). The lower lip is crescent shaped and sometimes weakly keratinised. The eyes are in the anterior half of the head. The morphometric characters are summarised in Table 1.

The dorsal fin is short and weakly ossified and more than 15 % of the length of its last unbranched ray is flexible. Its last unbranched ray is about as long as the head (Fig. 4). It usually has four unbranched and seven to 10 branched rays (Table 3). The anal fin usually has three unbranched and five or six branched rays (Table 4). Its length is rather variable in adult specimens. It reaches the base of the caudal fin in some specimens.

*Carasobarbus fritschii* has 30 to 39 scales in the lateral line (Table 5), usually 5.5 scales above the lateral line (Table 6), usually 4.5 or 5.5 scales below the lateral line (Table 7), and 14 to 20 scales around the least circumference of the caudal peduncle (Table 8). The scales are shown in Fig. 5.

The pharyngeal teeth count is 2.3.4-4.3.2 in two specimens, 2.3.4- in one specimen and -4.3.2 in eight specimens. Pharyngeal teeth are hooked at their tips (Fig. 6).

Live specimens are silvery and usually have a dark longitudinal band above the lateral line. Fins are hyaline to slightly orange (Fig. 17). Ethanol-preserved specimens are yellow-brown, the back is usually distinctly darker than the belly and flanks.

The maximum length observed in the material available is 180 mm SL.

*Carasobarbus fritschii* differs from all congeners except *Carasobarbus exulatus* and *Carasobarbus harterti* in having nine instead of 10 branched dorsal-fin rays. It differs from *Carasobarbus exulatus* in having 30 to 39 scales in the lateral line vs. 26 to 32 and modally 16 scales around the least circumference of the caudal peduncle vs. 12. It differs from *Carasobarbus harterti* in having a convex dorsal head profile and a last unbranched dorsal-fin ray that is weakly ossified and flexible for more than 15 % of its length vs. a straight dorsal head profile and a strongly ossified last unbranched dorsal-fin ray that is flexible in less than 15 % of its length.

#### Distribution.

**Figure 18.**
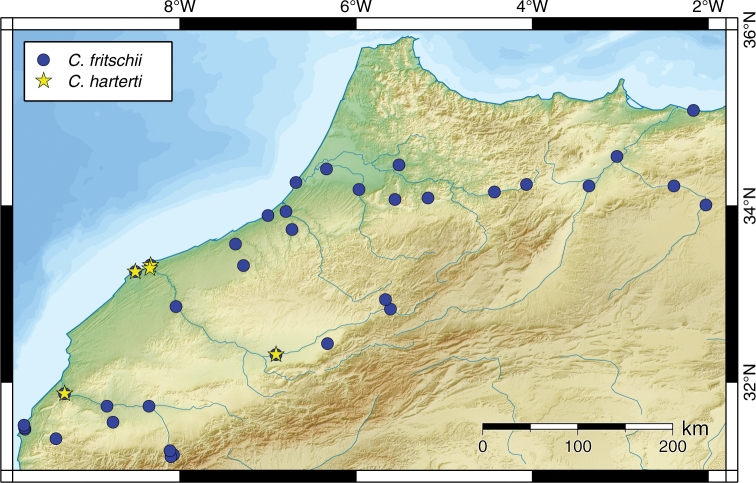
Map of the distribution of *Carasobarbus fritschii* and *Carasobarbus harterti*.

*Carasobarbus fritschii* is widespread and abundant in Northern and Central Morocco (Fig. 18). It occurs in the Oued al Maleh, Oued Bou Regreg, Oued Igrounzar, Oued Moulouya, Oued Oum er Rbia, Oued Sebou and Oued Tennsift drainage systems, and in numerous small coastal rivers. Most records are from Morocco, but one specimen is from the Oued Kiss in Algeria.

#### Habitats and biology.

*Carasobarbus fritschii* occurs in a wide range of running water courses and dam lakes.

#### Conservation status.

*Carasobarbus fritschii* is a hardy species and occurs in near-natural as well as heavily modified habitats. It is tolerant against pollution, damming and the presence of several exotic species (KB pers. obs.). The IUCN rates *Carasobarbus fritschii* as “Least Concern” and *Barbus paytonii* (which is treated as a junior synonym in this study) as “Vulnerable B2ab(iii)” (Crivelli 2006c, Crivelli 2006e). According to the latter assessment the population in the lower Oued Oum er Rbia is adversely affected by agricultural pollution (Crivelli 2006e).

#### Discussion.

*Carasobarbus fritschii* was described from the Oued Ksob as a member of the genus *Barbus* (Günther 1874). The same author described *Barbus rothschildi* from the Oued Oum er Rbia (Günther 1901). It is a junior synonym of *Carasobarbus fritschii*. One year later Günther (1902) described *Barbus riggenbachii* from Oued Oum er Rbia and Oued Talmest. It is a junior synonym of *Carasobarbus fritschii*. In the same year *Capoeta atlantica* and *Capoeta waldoi* were described from Oued Nfis (Boulenger 1902). These two species were placed into *Capoeta*, based on the keratinised lower lip that occurs in some specimens of *Carasobarbus fritschii*. Both are junior synonyms of *Carasobarbus fritschii*. *Barbus paytonii* was described from Oued Oum er Rbia (Boulenger 1911). It is a junior synonym of *Carasobarbus fritschii*. In the same publication Boulenger transferred *Capoeta waldoi* to the genus *Barbus*. The junior synonyms listed above were described, based on slight differences in mouth and lower lip shape or the degree of ossification of dorsal-fin rays. Sample sizes were usually very small. The examination of a large number of specimens revealed high variability and a continuous distribution of these characters. Boulenger (1919) transferred all species to the genus *Barbus* subgenus *Labeobarbus*, based on the possession of scales with parallel radii and an unserrated last unbranched dorsal-fin ray. Pellegrin (1919) listed the species in the genus *Barbus* but later (Pellegrin 1921, 1939) accepted the subgenus *Labeobarbus*. Pellegrin (1939) synonymised *Barbus riggenbachi* with *Barbus rothschildi* and did not list *Capoeta atlantica*. Karaman (1971) created the genus *Pseudotor* and synonymised *Capoeta atlantica* and *Capoeta waldoi* with *Pseudotor fritschii fritschii*. Fowler (1976) accepted all previously described species and transferred *Capoeta atlantica* and *Capoeta waldoi* to the genus *Varicorhinus*. Berrebi (1981) used the genus *Barbus* subgenus *Labeobarbus* and found no relevant differences between *Barbus fritschii* and *Barbus paytonii* in his morphometric and biochemical analysis. El Gharbi et al. (1993) highlighted the African distribution of the subgenus *Labeobarbus*. Doadrio (1994) and Tsigenopoulos et al. (2010) used *Labeobarbus*. Subsequent authors used the genus *Barbus* (Azeroual et al. 2000, Machordom and Doadrio 2001, Leggatt and Iwama 2003, Colli et al. 2009) or the provisional genus ‘*Barbus*’ (Borkenhagen et al. 2011). We transfer this species to the genus *Carasobarbus*, based on the possession of a smooth last unbranched dorsal-fin ray, modally nine branched dorsal-fin rays, six branched rays in the anal fin and shield-shaped scales with numerous parallel radii. Analysis of molecular genetic characters (Durand et al. 2002, Tsigenopoulos et al. 2010, KB unpublished data) support this decision.

The name of this species is frequently misspelled “*Barbus fritschi*”.

The ‘Catalog of Fishes’ lists SMF 636 and SMF 952 as types for *Carasobarbus fritschii* (Eschmeyer 2011). Both lots where collected by K. v. Fritsch and J. Rein in Oued Ksob in 1872, together with the types of ‘*Barbus*’ *reinii* Günther, 1874, *Luciobarbus nasus* (Günther, 1874) and the syntypes of *Carasobarbus fritschii*. SMF 636 contains seven specimens: one *Luciobarbus nasus*, one *Luciobarbus ksibi* (Boulenger, 1905), one ‘*Barbus*’ *reinii* and four *Carasobarbus fritschii*. SMF 952 contains eight specimens: two ‘*Barbus*’ *reinii* and six *Carasobarbus fritschii*. In the original description Günther (1874) did not state the number of type specimens on which he based the description of *Carasobarbus fritschii*, but in the same paper he described *Luciobarbus nasus* (as *Barbus nasus*), based on two specimens and ‘*Barbus*’ *reinii*, based on three specimens. It is likely that Günther never saw the lots SMF 636 and SMF 952, because all syntypes of *Luciobarbus nasus* and ‘*Barbus*’ *reinii* are in the BMNH. The collectors, K. v. Fritsch and J. Rein probably deposited these samples immediately in the SMF and we conclude that SMF 636 and SMF 952 are not part of the type series of *Carasobarbus fritschii*.

### Carasobarbus harterti

(Günther, 1901) comb. n.

http://species-id.net/wiki/Carasobarbus_harterti

Barbus harterti Günther 1901: 367.

#### Material.

**Type material.** Syntypes: BMNH 1901.7.26:4-5, 2, Morocco, Oued Oum er Rbia [33°19'40"N, 8°20'2"W], E. Hartert.

**Non-type material.** Oued Oum er Rbia drainage. BMNH 1902.7.28:27-33, 7; BMNH 1903.10.29:11-15, 8, Morocco, Oued Oum er Rbia [33°19'40"N, 8°20'2"W], F. Riggenbach. - BMNH 1903.7.1:5-7, 3, Morocco, Oued Oum er Rbia near El Jadida [33°15'18"N, 8°30'22"W], F. Riggenbach. - MNHN 1912-0089, 1; MNHN 1912-0090, 1; MNHN 1912-0091, 1; MNHN 1912-0092, 1; MNHN 1912-0093, 1, Morocco, Oued Oum er Rbia near Azemmour [33°17'22"N, 8°20'33"W], C. du Gast, 1912. - SMF 33366, 1; SMF 33368, 1; SMF 33370, 1, Morocco, Oued Oum er Rbia (32°18'53"N, 6°54'33"W), A. Azeroual et al., 9 Apr 2011.

Oued Tennsift drainage. BMNH 1902.7.28:34, 1, Morocco, Oued Talmest [31°52'15"N, 9°18'31"W], F. Riggenbach.

#### Diagnosis.

Two pairs of long barbels; 31 to 38 scales in the lateral line and 13 to 17 scales around the least circumference of the caudal peduncle; dorsal fin longer than anal fin and less than 15 % of the length of its last unbranched ray is flexible, dorsal profile of the head straight.

#### Description.

**Figure 19.**
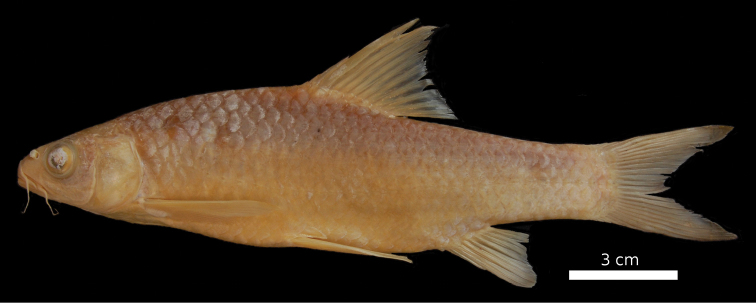
*Carasobarbus harterti*, syntype (BMNH 1901.7.26:4-5) from Oued Oum er Rbia, ^©^ The Natural History Museum, London.

**Figure 20.**
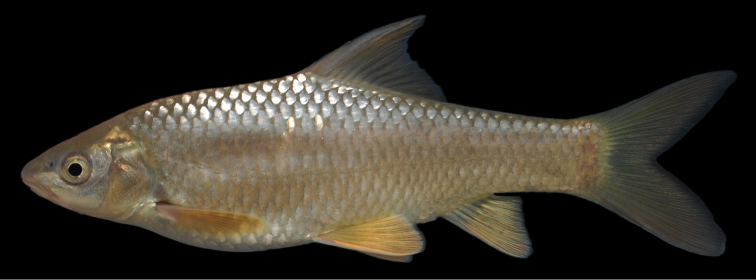
*Carasobarbus harterti*, live specimen from Oued Oum er Rbia.

The body is of moderate height and without a nuchal hump. The head is triangular with almost straight dorsal and ventral profile (Figs 19, 20). The head length is shorter than the body depth (Fig. 12). The mouth is subterminal with two pairs of long barbels (Table 2). The eyes are in the anterior half of the head and relatively big. The morphometric characters are summarised in Table 1.

The dorsal fin is long and strongly ossified and less than 15 % of the length of its last unbranched ray is flexible. Its last unbranched ray is as long as or longer than the head (Fig. 4). It usually has four unbranched and nine branched rays (Table 3). The anal fin usually has three unbranched and six or seven branched rays (Table 4). It does not reach the caudal fin origin.

*Carasobarbus harterti* has 31 to 38 scales in the lateral line (Table 5), usually 5.5 or 6.5 scales above the lateral line (Table 6), 4.5 to 6.5 scales below the lateral line (Table 7) and 13 to 17 scales around the least circumference of the caudal peduncle (Table 8). The scales are shown in Fig. 5.

The pharyngeal teeth count is -4.3.2 in four specimens examined. The pharyngeal teeth are hooked at their tips (Fig. 6).

Live specimens are silvery with an olive tinge and orange fins (Fig. 20). Ethanol-preserved specimens are yellow-brown, the back is darker than the belly and flanks.

The maximum length observed in the material examined is 250 mm SL.

*Carasobarbus harterti* differs from all congeners except *Carasobarbus exulatus* and *Carasobarbus fritschii* in having nine rather than 10 branched dorsal-fin rays. It differs from *Carasobarbus exulatus* in having 31 to 38 scales in the lateral line vs 26 to 32 and modally 16 scales around the least circumference of the caudal peduncle vs. 12. It differs from *Carasobarbus fritschii* in having a straight dorsal head profile and a last unbranched dorsal-fin ray that is strongly ossified and flexible for less than 15 % of its length vs. a convex dorsal head profile and a last unbranched dorsal-fin ray that is weakly ossified and flexible for more than 15 % of its length.

#### Distribution.

*Carasobarbus harterti* occurs in the rivers of the Oued Oum er Rbia and Tennsift drainage systems in Morocco (Fig. 18).

#### Habitats and biology.

*Carasobarbus harterti* is less common than *Carasobarbus fritschii* and inhabits only the lower and middle course of big rivers.

#### Conservation status.

The IUCN rates this species as “Vulnerable A2ace“ (Crivelli 2006d). The population has declined more than 30 % in the time from 1996 to 2006 due to urban, agricultural and industrial pollution (Crivelli 2006d).

#### Discussion.

*Carasobarbus harterti* was described from Oued Oum er Rbia as *Barbus harterti* (Günther 1901). Some authors placed this species in the genus *Barbus* subgenus *Labeobarbus* (Boulenger 1919, Pellegrin 1921) while others continued using the genus *Barbus* (Pellegrin 1919, 1939). Karaman (1971) synonymised it with *Carasobarbus fritschii*, but regarded it as a distinct subspecies. He incorrectly synonymised *Barbus rothschildi*, *Barbus riggenbachi* and *Barbus paytonii* with this subspecies and placed it in his newly erected genus *Pseudotor*. Subsequent authors did not accept Karaman’s proposal and continued using *Barbus* (Fowler 1976, El Gharbi et al. 1993, Azeroual et al. 2000, Leggatt and Iwama 2003, Colli et al. 2009, Borkenhagen et al. 2011) or proposed using *Labeobarbus* (Doadrio 1994, Tsigenopoulos et al. 2010). We transfer this species to the genus *Carasobarbus*, based on the possession of a smooth last unbranched dorsal-fin ray, nine branched dorsal-fin rays, six branched rays in the anal fin and shield-shaped scales with numerous parallel radii. Analysis of molecular genetic characters (Durand et al. 2002, Tsigenopoulos et al. 2010, KB unpublished data) support this decision.

### Carasobarbus kosswigi

(Ladiges, 1960)

http://species-id.net/wiki/Carasobarbus_kosswigi

Cyclocheilichthys kosswigi Ladiges 1960: 135.

#### Material.

**Type material.** Holotype of *Cyclocheilichthys kosswigi*: ZMH H 1148, Turkey, Batman Çayı [37°47'16"N, 41°0'51"E], C. Kosswig, IV.1939.

**Non-type material.** Tigris-Euphrates system. NMW 90369, 1, Turkey, Batman Çayı near Baschkaja [37°53'15"N, 41°7'56"E], V. Pietschmann, 15 Jul 1914. - NMW 90805, 1, Turkey, Gökçesu Çayı (37°45'N, 41°45'E), 26 Sep 1985. - ZMH 9548, 2, Turkey, Ceylanpınar [36°50'50"N, 40°3'0"E]. - SMF 33119, 1, Syria, Nahr al Khābūr at Al Ḩasakah [36°30'9"N, 40°44'52"E], F. Krupp. - SMF 30172, 1, Syria, Nahr al Khābūr near Tall Budayrī (36°24'N, 40°52'E), F. Krupp, 2–4 Nov 1986. - SMF 30173, 1, Syria, Nahr al Khābūr near Nahāb (36°23'N, 40°50'E), F. Krupp, 23–27 May 1989. - SMF 30174, 1, Syria, Nahr al Khābūr near Nahāb (36°23'N, 40°50'E), F. Krupp, 28 Sep–8 Oct 1988. - CMNFI 79-0290, 2, Iran, Qaşr-e Shīrīn (34°31'N, 45°35'E). - CMNFI 79-0289, 1, Iran, 25–30 km from Qaşr-e Shīrīn (34°28'N, 45°52'E). - BMNH 1974.2.22:1292-1296, 4; BMNH 1974.2.22:1281, 1, Iraq, Euphrates at Ḩadīthah [34°8'23"N, 42°22'41"E], 19 Oct 1953. - CMNFI 79-0275, 1, Iran, Rūdkhāneh-ye Kashgān, 2 km from Ma‘mūlān (33°25'N, 47°58'E). - SMF 33129, 3, Iran, Rūdkhāneh-ye Karkheh at Pol-e Dokhtar (33°9'36"N, 47°43'12"E), N. Alwan et al., 3 Mar 2008. - ZM-CBSU 4153, 1; ZM-CBSU 4154, 1, Iran, Rūdkhāneh-ye Dez at Dezfūl [32°22'57"N, 48°24'7"E], F. Bossaghzadeh, 8 Jun 2005.

#### Diagnosis.

Two pairs of barbels; 32 to 38 scales in the lateral line, usually 14 to 16 scales around the least circumference of the caudal peduncle; last unbranched dorsal-fin ray markedly longer than head; mouth narrow, lower lip spatulate and median lobe present.

#### Description.

**Figure 21.**
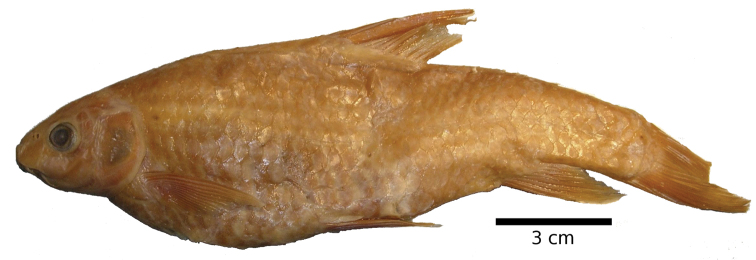
*Carasobarbus kosswigi*, holotype (ZMH 1148) from Batman Çayı.

**Figure 22.**
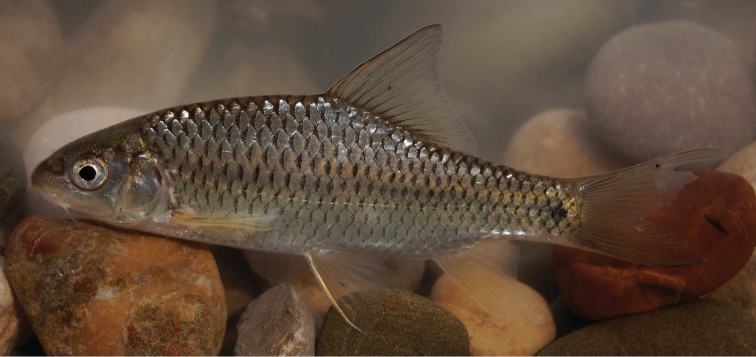
*Carasobarbus kosswigi*, live specimen from Rūdkhāneh-ye Karkheh.

Body moderately high, laterally compressed and without a nuchal hump. The greatest body depth is at the point of the origin of the dorsal fin. The ventral profile of the head is straight, its dorsal profile has a slight to pronounced hump near the nostrils (Figs 21, 22). The head is short and narrow. The mouth is inferior. The maximum body depth is bigger than the head length (Fig. 12). The lips are comparatively thick and the lower jaw is narrow with a sharp horny sheath and a median lobe. The two pairs of barbels (Table 2) are stout and the anterior pair is quite long. The eyes are rather high in the middle of the head and rather small. The morphometric characters are summarised in Table 1.

The dorsal fin is long and usually has four unbranched and nine or 10 branched rays (Table 3). The last unbranched ray is long and well ossified; only the tip is flexible. It is considerably longer than the head (Fig. 4). The anal fin usually has three unbranched rays and six branched rays (Table 4). Its base is long. The bases of the dorsal and anal fin have a sheath of scales.

There are 32 to 38 scales in the lateral line (Table 5), 5.5 to seven scales above the lateral line (Table 6), 4.5 to 6.5 scales below the lateral line (Table 7) and (12) 14 to 16 scales around the least circumference of the caudal peduncle (Table 8). The scales are shown in Fig. 5.

The pharyngeal teeth count is 2.3.5-5.3.2 in seven specimens, 2.3.5- in one specimen and -4.3.2 in one specimen. The pharyngeal teeth are hooked at their tips (Fig. 6).

Live specimens are silvery. The back is darker than the belly, which is almost white (Fig. 22). Fixed specimens are yellow-brown and some have a darker back.

*Carasobarbus kosswigi* differs from all congeners, except *Carasobarbus sublimus*, by having a spatulate lower jaw with a median lobe on the lower lip vs. a crescent-shaped lower jaw and a lower lip without median lobe. It differs from *Carasobarbus sublimus* by having 32 to 38 scales in the lateral line vs. 27 to 29 and modally 14 scales around the least circumference of the caudal peduncle vs. 12 and by having a longer and more ossified last unbranched ray in the dorsal fin.

#### Distribution.

*Carasobarbus kosswigi* occurs in the Euphrates-Tigris system (Fig. 7).

#### Habitats and biology.

*Carasobarbus kosswigi* is rare, inhabits fast-flowing reaches of rivers and feeds on small animals (Krupp and Schneider 2008). The maximum length is about 150 mm SL and this species has no economic importance (Krupp and Schneider 2008).

#### Conservation status.

Little information is available, but because this species is dependent on fast-flowing water, it is probably impacted by the construction of dams.

#### Discussion.

*Carasobarbus kosswigi* was described from the Batman Çayı and placed in the genus *Cyclocheilichthys* (Ladiges 1960). Karaman erected the new genus *Kosswigobarbus* for this species (Karaman 1971). Coad gave a detailed re-description of this species and transferred it to the genus *Barbus* (Coad 1982). *Kosswigobarbus* was revalidated (Ekmekçi and Banarescu 1998) and sometimes used as a subgenus of *Barbus* (Tsigenopoulos et al. 2010). Later the species was placed in *Carasobarbus* (Borkenhagen et al. 2011).

*Carasobarbus kosswigi* is paraphyletic with respect to *Carasobarbus sublimus* (Borkenhagen et al. 2011).

### Carasobarbus luteus

(Heckel, 1843)

http://species-id.net/wiki/Carasobarbus_luteus

Systomus luteus Heckel 1843: 1161.
Systomus albus Heckel 1843: 1163.
Systomus albus var. *alpina*Heckel 1847: 257.
Barbus parieschanica Wossughi et al. 1983: 34.

#### Material.

**Type material.** Nahr Quwayq basin. Paralectotypes of *Systomus luteus*: NMW 54248, 1; NMW 54250:1-2, 2; NMW 54254:1-3, 3; SMF 6784, 1, Syria, Nahr Quwayq near Aleppo [36°12'10"N, 37°9'31"E], T. Kotschy, 17 May 1842.

Syntypes of *Systomus albus*: NMW 53674-53677, 4; NMW 53680, 1; SMF 812, 1, Syria, Nahr Quwayq near Aleppo [36°12'10"N, 37°9'31"E], T. Kotschy, 18 May 1842.

Rūd-e Mand basin. Syntypes of *Systomus albus alpina*: NMW 53678, 5; NMW 53679:1-2, 2; NMW 53681:1-2, 2, Iran, Rūdkhāneh-ye Qarah Āghāj near Shīrāz [29°31'3"N, 52°15'0"E], 2 Jan 1844.

Rūdkhāneh-ye Ḩelleh basin. Syntypes of *Systomus albus alpina*: NMW 53682:1-2, 2, Iran, Daryācheh-ye Parīshān [29°31'7"N, 51°47'47"E].

Tigris-Euphrates system. Lectotype of *Systomus luteus* (by present designation): NMW 54253:2, Iraq, Tigris near Mosul [36°20'6"N, 43°7'8"E], T Kotschy, 10 Apr 1843.

Paralectotypes of *Systomus luteus*: NMW 54247:1-2, 2; NMW 54249, 1; NMW 54253:1, 1; NMW 54255:1-2, 2; NMW 80043, 2 same data as lectotype.

Syntype of *Systomus albus*: NMW 91400, 1, Iraq, Tigris near Mosul [36°20'6"N, 43°7'8"E], 11 Apr 1843.

Unknown drainage system. Paralectotype of *Systomus luteus*: NMW 10827, 1, Syria, “Damascus”, T. Kotschy, 1837.

**Non-type material.** Daryācheh-ye Mahārlū basin. CMNFI 79-0047, 1, Iran, source of Ab-e Paravan marshes 19.9 km from Shīrāz University [29°36'N, 52°32'E]. - FSJF 2232, 2, Iran, Pirbano spring about 10 km south of Shīrāz (29°31'8"N, 52°27'56"E), A. Abdoli and J. Freyhof, 21 Apr 2007. - ZM-CBSU 3439, 1; ZM-CBSU 3449, 1; ZM-CBSU uncatalogued, 1, Iran, Pol-e Berenji, southwest of Shīrāz [29°27'30"N, 52°32'0"E], H. R. Esmaeili et al. - CMNFI 79-0347, 1, Iran, Solţānābād marshes near Pol-e Berenji (29°27'30"N, 52°32'0"E).

Orontes basin. MNHN 1977-0255, 1, Syria, Orontes, Gruvel, 1929, only one of two specimen examined. - MNHN 1977-0257, 1, Syria, Orontes, Gruvel, 1930. - SMF 24341, 1, Syria, Orontes at Jisr ash Shughūr (35°48'N, 36°19'E), F. Krupp, 21 Mar 1979 (aberrant specimen).

Rūd-e Mand basin. CMNFI 79-0206, 1, Iran, Qanat 41 km from Estahbān on road to Kharāmeh (29°12'N, 53°40'E). - CMNFI 79-0160, 1, Iran, cement pool near spring along road to Neyrīz (29°9'N, 53°37'E). - ZM-CBSU 4934-4942, 9, Iran, Dareh Daarveshan between Rudbal and Simakan (28°39'10"N, 52°2'27"E), H. R. Esmaeili et al. - ZM-CBSU 101-103, 3; ZM-CBSU 110, 1; ZM-CBSU uncatalogued, 1, Iran, Rūdkhāneh-ye Sīmakān near Jahrom [28°30'0"N, 53°33'38"E], H. R. Esmaeili et al.

Rūdkhāneh-ye Ḩelleh basin. CMNFI 79-0026, 1, Iran, Rūdkhāneh-ye Shāhpūr near Shahr-e Tārīkhī-ye Neyshābūr (29°47'N, 51°35'E). - ZM-CBSU 5180-5190, 10; ZM-CBSU 5192, 1, Iran, Kāzerūn, Sarab Dokhtar [29°37'10"N, 51°39'15"E], H. R. Esmaeili et al. - ZM-CBSU 6508-6517, 10; ZM-CBSU 6574, 1; ZM-CBSU 6602-6607, 6; ZM-CBSU 6610, 1; ZM-CBSU 6614+6615+6617-6619, 5; ZM-CBSU uncatalogued, 12, Iran, Daryācheh-ye Parīshān [29°31'7"N, 51°47'47"E], H. R. Esmaeili et al. - CMNFI 79-0240, 2; CMNFI 79-0304, 3, Iran, Daryācheh-ye Parīshān (29°31'N, 51°50'E). - CMNFI 79-0125, 1, Iran, Rūdkhāneh-ye Dālakī near Dālakī (29°28'N, 51°21'E). - ZM-CBSU 2650-2651, 2; ZM-CBSU 2654-2655, 2, Iran, spring at Palangī Dādīn, near Kāzerūn, Rūdkhāneh-ye Dālakī [29°25'20"N, 51°43'54"E], H. R. Esmaeili et al.

Rūdkhāneh-ye Kol basin. ZM-CBSU 3219-3229, 11; ZM-CBSU 3252-3260, 9, Iran, Golabi spring north of Dārāb [28°47'15"N, 54°22'19"E], H. R. Esmaeili et al. - FSJF 2253, 6, Iran, Golabi spring 35 km north of Dārāb (28°47'15"N, 54°22'19"E), A. Abdoli and J. Freyhof, 21 Apr 2007. - CMNFI 79-0155, 1, Iran, spring at Gavanoo, east of Ḩasanābād [28°47'N, 54°22'E]. - CMNFI 79-0154, 2, Iran, Korsia village on Dārāb-Fasā road (28°45'30"N, 54°24'0"E). - ZM-CBSU 5622-5626, 5, Iran, Tang-e Khūr near Lār [27°36'N, 54°17'E], H. R. Esmaeili et al.

Rūdkhāneh-ye Naband basin. CMNFI 79-0187, 10, Iran, stream and pools at Sarkhūn, Rūdkhāneh-ye Sarzeh (27°23'30"N, 56°26'0"E).

Tigris-Euphrates system. SMF 30208, 1, Turkey, Tigris at Diyarbakır (37°53'N, E40°14’), R. Kinzelbach, 1982. - SMF 30176, 11, Syria, Nahr al Khābūr at Ra’s al ‘Ayn (36°51'N, 40°4'E), F. Krupp, 24–26 May 1989. - SMF 30186, 12, Syria, ‘Ayn Sālūba and ‘Ayn Hamza near Ra’s al ‘Ayn (36°51'N, 40°4'E), F. Krupp, 3 Oct 1988. - SMF 30200, 2, Syria, ‘Ayn Sālūba at Ra’s al ‘Ayn (36°51'N, 40°4'E), F. Krupp, 3 Oct 1988. - SMF 30190, 7, Syria, Nahr al Khābūr 2 km East of Tall Junaydīyah (36°44'N, 40°6'E), F. Krupp, 26 May 1989. - SMF 30197, 2, Syria, Nahr al Khābūr 2 km East of Tall Junaydīyah (36°44'N, 40°6'E), F. Krupp, 5 Oct 1988. - SMF 30179, 3, Syria, Nahr al Khābūr at Tall ʿAtaš (36°42'N, 40°11'E), F. Krupp, 26 May 1989. - SMF 30188, 3, Syria, Nahr al Khābūr at Tall ʿAtaš (36°42'N, 40°11'E), F. Krupp, 6 Oct 1988. - SMF 31317, 1; SMF 33139, 7, Syria, Nahr al Khābūr at Tall Tamr (36°39'7"N, 40°21'51"E), N. Alwan et al., 29 Oct 2008. - SMF 30199, 1, Syria, Nahr al Khābūr at Tall Naşrī (36°37'N, 40°23'E), F. Krupp, 6–7 Oct 1988. - SMF 30178, 1; SMF 30202, 10, Syria, Nahr al Khābūr near Tall Bāz (36°35'N, 40°27'E), F. Krupp, 7 Oct 1988. - SMF 30184, 1; SMF 30193, 3, Syria, Nahr al Khābūr at Tall Bāz (36°35'N, 40°27'E), F. Krupp, 26 May 1989. - SMF 30181, 1; SMF 30192, 3, Syria, Nahr al Khābūr at Tall Umm al Māʿaz (36°34'N, 40°35'E), F. Krupp, 27 May 1989. - SMF 30183, 3, Syria, Nahr al Khābūr at Umm al-Māʿaz (36°34'N, 40°35'E), F. Krupp, 7 Oct 1988. - SMF 30182, 2, Syria, Nahr al Khābūr at Al Ḩasakah (36°30'N, 40°44'E), F. Krupp, 27 May 1989. - SMF 30195, 1, Syria, Nahr al Khābūr at Al Ḩasakah (36°30'N, 40°44'E), F. Krupp, 7 Oct 1988. - SMF 30185, 1; SMF 30213, 6, Syria, Nahr al Khābūr and Wādī Furātī at Tall Tayyiǧ (36°26'N, 40°52'E), F. Krupp, 8 Oct 1988. - SMF 30189, 4, Syria, Nahr al Khābūr at Baḩrat Khātūnīyah (36°24'N, 41°13'E), F. Krupp, 23–24 May 1989. - SMF 30214, 5, Syria, Nahr al Khābūr at Tall Budayrī (36°24'N, 40°49'E), F. Krupp, 26 Sep–8 Oct 1988. - SMF 30206, 7, Syria, Nahr al Khābūr at Tall Budayrī (36°24'N, 40°52'E), F. Krupp, 2–4 Nov 1986. - SMF 30177, 3, Syria, Nahr al Khābūr at Nahāb (36°23'N, 40°50'E), F. Krupp, 28 Sep–8 Oct 1988. - SMF 30201, 23, Syria, Nahr al Khābūr at ‘Ayn Ţābān (36°22'N, 40°50'E), F. Krupp, 28 Sep 1988. - SMF 30191, 2, Syria, Nahr al Khābūr at mouth of Wādī ar Raml (36°15'N, 40°48'E), F. Krupp, 8 Oct 1988. - SMF 30196, 1, Syria, Nahr al Khābūr at Umm Rukaybah (36°8'N, 40°42'E), F. Krupp, 8 Oct 1988. - SMF 30194, 3, Syria, Nahr al Khābūr at Ash Shaddādah (36°4'N, 40°44'E), F. Krupp, 9 Oct 1988. - SMF 31316, 1; SMF 33138, 2, Syria, Nahr al Khābūr at Ash Shaddādah (36°3'46"N, 40°44'30"E), N. Alwan et al., 28 Oct 2008. - SMF 33152, 6, Syria, Jisr Shānīn (36°3'4"N, 39°5'10"E), F. Krupp and W. Schneider, 19 Aug 1980. - SMF 31308, 1, Syria, Mamlaḩat al Jabbūl (36°3'36"N, 37°33'1"E), N. Hamidan, 23 Jun 2008. - SMF 28707, 18, Syria, Euphrates down stream Buḩayratt al Asad (35°51'48"N, 39°0'34"E), R. Beck, Jun 1998. - SMF 30198, 2, Syria, Nahr al Khābūr at Tall ash Shaykh Ḩamad (35°37'N, 40°45'E), F. Krupp, 21 Sep–14 Oct 1988. - SMF 30204, 1; SMF 30205, 4, Syria, Nahr al Khābūr at Tall ash Shaykh Ḩamad (35°37'N, 40°45'E), F. Krupp, 20 Oct–9 Nov 1986. - SMF 33140, 1; SMF 33141, 37, Syria, Euphrates at Harmūshīyah (35°35'52"N, 39°51'25"E), N. Alwan et al., 31 Oct 2008. - SMF 30203, 2, Syria, Nahr al Khābūr 8 km South of Tall ash Shaykh Ḩamad (35°33'N, 40°43'E), F. Krupp, 24 Oct 1986. - SMF 28737, 5, Syria, Euphrates between Ḩalabīyah-Zalābīyah and Dayr az Zawr, R. Beck, Jun 1998. - SMF 28630, 3, Syria, Euphrates upstream Dayr az Zawr (35°31'N, 39°54'E), R. Beck, 23 May 1998. - SMF 28674, 41, Syria, Euphrates upstream Dayr az Zawr [35°31'N, 39°54'E], R. Beck, 30 May 1998. - SMF 33153, 1, Syria, Nahr al Khābūr at Aş Şuwar (35°30'N, 40°38'E), F. Krupp, 15 Mar 1979. - SMF 31315, 1; SMF 33137, 1, Syria, Nahr al Khābūr at Ghawat (35°28'51"N, 40°39'54"E), N. Alwan et al., 28 Oct 2008. - SMF 30187, 2, Syria, Nahr al Khābūr near Ḩarījīyah (35°27'N, 40°38'E), F. Krupp, 10 Oct 1988. - SMF 30180, 5, Syria, Nahr al Khābūr at Mashikh (35°14'N, 40°31'E), F. Krupp, 10 Oct 1988. - SMF 28663, 6, Syria, Euphrates at Qal‘at aş Şāliḩīyah (Dura Europos) [34°45'0"N, 40°43'30"E], R. Beck, 28 May 1998. - SMF 28758, 2, Syria, Euphrates at Abū Kamāl at mouth of Wādī Ratqah [34°26'45"N, 40°56'0"E], R. Beck, 9 Jul 1998. - NMW 93019:1-2, 2, Iraq, Tigris at Baghdād [33°20'26"N, 44°24'3"E], V. Pietschmann, Aug 1910. - SMF 33127, 4, Iran, Rūdkhāneh-ye Bālārūd (32°35'19"N, 48°17'11"E), N. Alwan et al., 3 Mar 2008. - BMNH 1980.8.28:6, 1, Iran, Rūdkhāneh-ye Dez at Dezfūl [32°25'N, 48°13'E]. - SMF 33125, 1, Iran, Rūdkhāneh-ye Dez at Dezfūl (32°22'40"N, 48°22'58"E), N. Alwan et al., 2 Mar 2008. - SMF 33121, 5, Iran, Rūdkhāneh-ye Dez at Dezfūl (32°21'49"N, 48°21'28"E), K. Borkenhagen et al., 3 Nov 2006. - SMF 17303, 1, Iraq, Hawr al Ḩammār (30°50'N, 47°10'E), L. A. J. Al-Hassan, 1986. - SMF 30211, 1, Iraq, ‘Ayn Zālah 50 km west of Mosul, Z. Rahemo, 1990.

Unknown drainage system. SMF 33120, 2, Syria, fish market in Damascus (reported to be from Buḩayratt Ar Rastan [34°56'N, 36°44'E] in Orontes drainage), F. Krupp. - CMNFI 79-0687, 4, Iran, Shīrāz bazar (probably from Rūd-e Mand basin or Daryācheh-ye Mahārlū basin).

**Figure 23.**
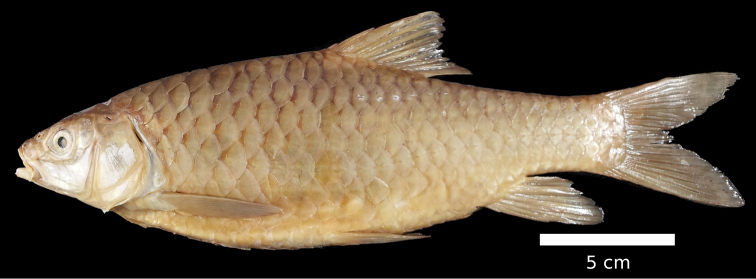
*Carasobarbus luteus*, lectotype (NMW 54253:2) from Tigris near Mosul, ^©^ Naturhistorisches Museum Wien, photo E. Lavergne.

The lectotype (NMW 54253:2) is a specimen of 211 mm SL, collected in the Tigris near Mosul on 10 Apr 1843 by T. Kotschy (Fig. 23). It has four unbranched and 10 branched rays in the dorsal fin, three unbranched and six branched rays in the anal fin, 27 scales in the lateral line and one pair of barbels. A bigger specimen (216 mm SL) from the same lot (NMW 54253:1) was not selected as lectotype, because it is atypical in having 11 branched rays in the dorsal fin and two pairs of barbels. The designation of a lectotype became necessary to fix the type locality of *Systomus luteus* (see Discussion).

#### Diagnosis.

One pair of barbels; 25 to 33 scales in the lateral line, and typically 12 scales around the least circumference of the caudal peduncle; last unbranched ray of the dorsal fin about as long as the head or slightly shorter.

#### Description.

Specimens from Rūdkhāneh-ye Naband basin were excluded from this species description (see below).

**Figure 24.**
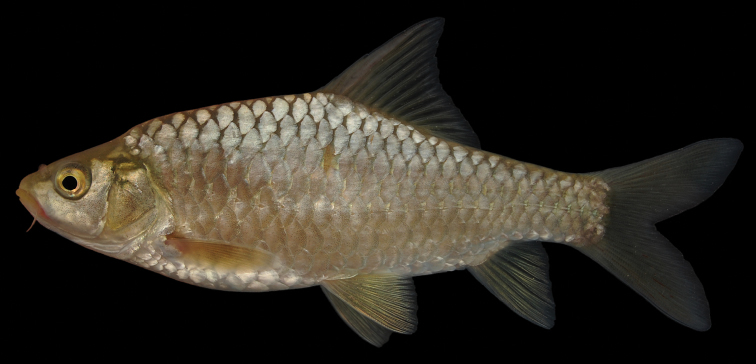
*Carasobarbus luteus*, live specimen from Nahr al Khābūr.

The dorsal profile is convex up to the origin of the dorsal fin and a nuchal hump is present in specimens longer than about 100 mm SL. This species has a high back and caudal peduncle (Figs 23, 24). The ventral profile of the head is convex, its dorsal profile is almost straight to convex and has a hump near the nostrils in juvenile specimens. The mouth is sub-terminal. The barbels are short and stout. The maximum body depth is usually greater than the head length (Fig. 12). Usually one pair of barbels is present, but about 10 % of the specimens have two pairs of barbels (Table 2). The eyes are at the back of the anterior half of the head. They are big and slightly protuberant. The morphometric characters are summarised in Table 1.

The dorsal fin usually has four unbranched and eight to 11 branched rays (Table 3). In specimens from the Tigris-Euphrates drainage system the last unbranched ray of the dorsal fin is strong with only the tip being flexible and it is about as long as the head. It is shorter and less ossified in Iranian populations (Fig. 4). The anal fin usually has three unbranched rays and five to seven branched rays (Table 4).

There are 25 to 33 scales in the lateral line (Table 5), 3.5 to 6 scales above the lateral line (Table 6), 3 to 5.5 scales below the lateral line (Table 7) and 10 to 13 scales around the least circumference of the caudal peduncle (Table 8). The scales are shown in Fig. 5.

The pharyngeal teeth count is 2.3.5-5.3.2 in 26 specimens, 2.3.4-5.3.2 in two specimens, 2.3.5-4.3.2 in one specimen, 2.3.5-5.3.3 in one specimen, 1.3.5-5.3.2 in one specimen, 2.3.5- in one specimen and 2.3.4- in one specimen. The pharyngeal teeth are hooked at their tips (Fig. 6).

Live specimens are silvery to olive and sometimes have yellowish fins (Fig. 24). Ethanol-preserved specimens are light yellowish brown to grey. In most cases the back is darker than the rest of the body. Some of the lighter coloured specimens have a salmon hue, others are silvery. The fins are yellowish brown to grey. Juveniles have a dark spot on the sides of the caudal peduncle.

*Carasobarbus luteus* from Ḩelleh, Kol, Mahārlū and Mand populations: The last unbranched ray of the dorsal fin is shorter and less well ossified. It is pronouncedly shorter than the head (Fig. 4). The mouth is wider and the body is not as high-backed as in specimens from the Tigris-Euphrates system (Fig. 12).

**Figure 25.**
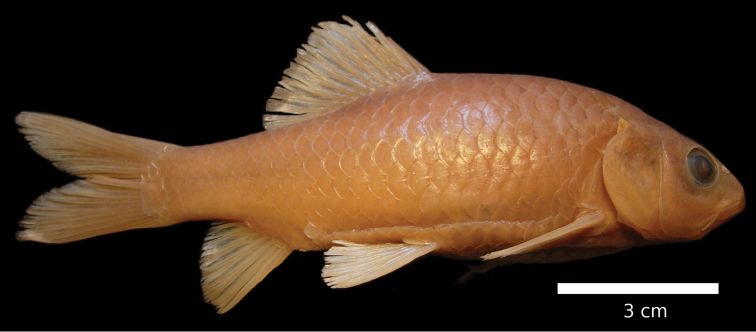
*Carasobarbus luteus*, specimen (CMNFI 79-0187) from Rūdkhāneh-ye Sarzeh.

*Carasobarbus luteus* from Rūdkhāneh-ye Naband basin: In this population all specimens examined had two pairs of barbels (Table 2). The anterior pair is longer than in specimens from Tigris-Euphrates system with two pairs. The last unbranched ray in the dorsal fin is considerably shorter than the head (Fig. 4) and comparatively weak. Compared with specimens from Tigris-Euphrates system, the dorsal and ventral fins tend to be slightly further away from the head. The head is longer and the body not as high backed as in specimens from Tigris-Euphrates system (Fig. 12). The general body shape (Fig. 25) resembles that of *Carasobarbus apoensis* and *Carasobarbus canis*. Some of the gill rakers are y-shaped in the largest specimen examined.

*Carasobarbus luteus*, except the population from Rūdkhāneh-ye Naband, differs from all congeners, except *Carasobarbus apoensis*, in having one instead of two pairs of barbels. It differs from *Carasobarbus apoensis*, *Carasobarbus canis*, *Carasobarbus chantrei*, *Carasobarbus fritschii*, *Carasobarbus harterti* and *Carasobarbus kosswigi* in modally having 28 scales in the lateral line vs. 30, 32, 34, 34, 34 and 33 respectively. It differs from *Carasobarbus kosswigi* and *Carasobarbus sublimus* in having a crescent-shaped lower lip without median lobe vs. a spatulate lower lip with median lobe and from *Carasobarbus exulatus*, *Carasobarbus fritschii* and *Carasobarbus harterti* in modally having 10 rather than nine branched dorsal-fin rays. All populations, except the one from Rūdkhāneh-ye Naband differ from *Carasobarbus apoensis* in having a shorter head and a higher back. The population from Rūdkhāneh-ye Naband is very similar to *Carasobarbus apoensis* in body shape, but differs in having two as compared to one pair of barbels.

#### Distribution.

*Carasobarbus luteus* has a much greater range than any of its congeners and its distribution area is fragmented, resulting in several isolated populations. It is widespread all over the Tigris-Euphrates drainage system, and occurs in the rivers of south-western Iran (Fig. 7). The Nahr al Quwayq population, from one of the sites of the type locality, is probably extirpated due to drought and pollution (Krupp 1980, Krupp 1983b). There are only few, mostly older, records from the Orontes (Krupp 1985c, Krupp 1987). During recent fieldwork *Carasobarbus luteus* was not found there. Because *Carasobarbus chantrei* is still widespread and abundant in many parts of the Orontes, it is unlikely that *Carasobarbus luteus* disappeared due to habitat degradation. It might have been driven out by competition with *Carasobarbus chantre* i or records were based on misidentifications or mislabelled specimens. One specimen (NMW 10827) is reported from Damascus. Because *Carasobarbus luteus* does not occur in the Damascus basin and it is highly unlikely that it ever occurred there, the origin of this specimen is unclear.

#### Habitats and biology.

*Carasobarbus luteus* is mainly herbivorous. It feeds on algae, aquatic plants, detritus and small invertebrates, the main feeding period is at noon, but food is also taken at night (Naama and Muhsen 1986). The intestine is long (Ali 1986). The maximum size is 38 cm total length and 750 g, but normally they are smaller than 35 cm and weight less than 500 g (Ahmed 1982). They reach maturity at the age of one or two years and at a size of about 14 cm; the spawning period is June and July in the Tigris-Euphrates system, the eggs are spawned among reeds, roots or other aquatic vegetation and fecundity is high (Al Hazzaa and Hussein 2003a).

This species can tolerate saline waters to some degree (Al-Hassan and Muhsin 1986, Mohamed et al. 1993) and is of commercial importance due to its size and abundance (Ahmed 1982, Barak and Mohamed 1983, Krupp and Schneider 2008).

There are attempts on aquaculture of this species. The stickiness of the eggs can be lowered by several chemical treatments for this purpose (Al Hazzaa and Hussein 2003b). During spawning males get reddish brown in the anterior part of the body and greenish at the caudal peduncle while females are less colourful (Al Hazzaa and Hussein 2003a). Males can produce series of sharp clicking noises which do not seem to be associated with aggressive behaviour (Al Hazzaa and Hussein 2003a).

Larvae hatch at 64 degree-days in well oxygenated water and the eyes are still without pigments at this stage. The development is similar to that of other cyprinids (Al Hazzaa and Hussein 2003a). Ahmed et al. (1984) studied the reproductive biology of *Carasobarbus luteus*.

**Conservation status.** *Carasobarbus luteus* is widespread and abundant in the Tigris-Euphrates system. Peripheral populations, like those in smaller Iranian rivers and the Nahr al Quwayq in Syria are more threatened or have already been extirpated (see above).

#### Discussion.

*Carasobarbus luteus* was described as *Systomus luteus* by Heckel (1843). Heckel (1843) listed Orontes, Tigris, Aleppo and Mosul as type localities. As all but one of the type specimens are either from the Tigris-Euphrates system or from the Nahr al Quwayq and Aleppo is located on the Nahr al Quwayq and not on the Orontes, Heckel may have confused these two rivers. One of the type specimens (NMW 10827) is from “Damascus” and can not be attributed to any of the relevant drainage systems. By designating NMW 54253:2 as lectotype we fix the Tigris near Mosul as type locality for *Systomus luteus*. The same confusion exists for the type localities of *Systomus albus*, which was also described from Tigris and Orontes in the same publication. A few years later *Systomus albus* var. *alpina* was described from the Daryācheh-ye Parīshān (Heckel 1847). These three taxa where later synomymised and placed in the genus *Barbus* (Günther 1868). Sauvage (1882, 1884) accepted *Carasobarbus luteus* and *Carasobarbus albus* as valid species and transferred them to the genus *Barynotus*. Later, both species where synonymised again and transferred to the genus *Barbus*, subgenus *Puntius* (Misra 1947) or the genus *Puntius* (Menon 1956). Ladiges (1960) synonymised both species under the name *Barynotus albus*. Because Günther (1868) had previously selected *luteus* as the valid species name, he is to be considered the first revising author and Ladiges’ action is not valid. Kähsbauer (1963) lists the species under two different generic names: *Barbus* (as *Barbus luteus*) and *Systomus* (as *Systomus albus* var. *alpina*). Karaman (1971) erected the new genus *Carasobarbus* for this species. This met mixed acceptance. While some authors accepted the new taxonomic position (e.g. Wossughi 1978, Bianco and Banarescu 1982, Ahmed et al. 1984, Naama and Muhsen 1986), others did not embrace it (e.g. Banister and Clarke 1977, Krupp 1985a, c, Coad 1995, Coad 1996) until the revision by Ekmekçi and Banarescu (1998). Fowler (1976) placed *Carasobarbus luteus* in the genus *Barbellion*. Tsigenopoulos et al. (2010) used *Barbus* subgenus *Carasobarbus*. *Barbus parieschanica* was described from Daryācheh-ye Parīshān (Wossughi et al. 1983). In the same publication the species name is also spelled *B. parschanica*, but *Barbus parieschanica* is probably the intended spelling (Coad 1995). Coad (1995) as the first revising author fixed *Barbus parieschanica* as the correct original spelling. *Barbus parieschanica* is a synonym of *Carasobarbus luteus*. The ‘Catalog of Fishes’ lists RMNH 2463 as possible syntype of *Systomus luteus* and RMNH 2464 of *Systomus albus* var. *alpina* (Eschmeyer 2011). We did not examine these specimens.

We do not think that the population at Rūdkhāneh-ye Naband should be elevated to specific rank, because the number of specimens available is too low. We provisionally consider it an atypical population of *Carasobarbus luteus* that might have been affected by bottleneck effects and accelerated morphological change, due to the restricted size and extreme conditions (high salinity and temperature) of its habitat. It would be very interesting to collect more samples for morphological studies and molecular sequence analysis.

In spite of some morphometric differences, *Carasobarbus luteus* populations of Tigris-Euphrates system and Iran belong to the same species (Borkenhagen et al. 2011); specimens from Rūdkhāneh-ye Naband were not included in that study.

*Carasobarbus luteus* and *Carasobarbus apoensis* are closely related to each other (KB, unpublished data) and *Carasobarbus apoensis* might be the ecologically specialised sister species of *Carasobarbus luteus*, that is adapted to the environmental conditions of the wadi ecosystems of the Al Ḩijāz mountains.

### Carasobarbus sublimus

(Coad & Najafpour, 1997)

http://species-id.net/wiki/Carasobarbus_sublimus

Barbus sublimus Coad and Najafpour 1997: 274.

#### Material.

**Type material.** Holotype of *Barbus sublimus*: CMNFI 1995-0009, Iran, Rūdkhāneh-ye A‘lā near Pol-e Tīghen (31°23'30"N, 49°53'0"E), B. W. Coad et al., 20 Sep 1995, not examined.

Paratypes of *Barbus sublimus*: CMNFI 95-0009a, 1, same data as holotype. - CMNFI 95-0010, 1, same data as holotype, not examined. - CMNFI 95-0011, 3, Iran, Rūdkhāneh-ye A‘lā near Pol-e Tīghen (31°23'30"N, 49°53'0"E), G. Eskanderi, Dec 1994, only one specimen examined.

**Non-type material.** Rūdkhāneh-ye Kashgān. CMNFI 79-0277, 1, Iran, Rūdkhāneh-ye Kashgān at Harpul Kashkow, 50 km from Khorramābād (33°30'0"N, 47°59'30"E), K. Evans and H. Assadi, 5 Jul 1977.

Rūdkhāneh-ye Zohreh drainage. ZM-CBSU 5781-5786, 6, Iran, Rūdkhāneh-ye Fahlīān at Nūrābād [30°6'51"N, 51°31'18"E], H. R. Esmaeili et al. - SMF 33117, 3, Iran, Rūdkhāneh-ye Fahlīān (30°11'10"N, 51°31'14"E), K. Borkenhagen et al., 29 Nov 2007. - SMF 33118, 6, Iran, Rūdkhāneh-ye Fahlīān (30°11'9"N, 51°31'15"E), N. Alwan et al., 29 Feb 2008.

#### Diagnosis.

Two pairs of barbels; 27 to 29 scales in the lateral line, 12 scales around the least circumference of the caudal peduncle; last unbranched dorsal-fin ray about as long as the head; mouth narrow, lower jaw spatulate and median lobe present on lower lip.

#### Description.

**Figure 26.**
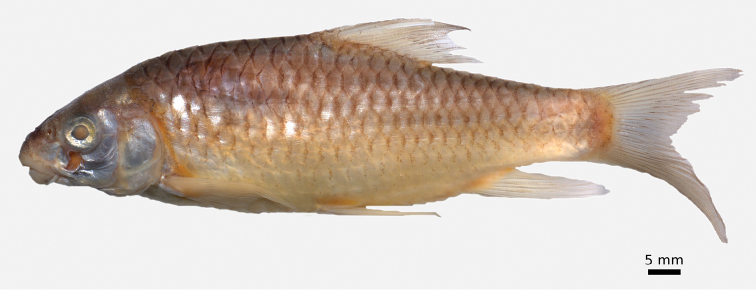
*Carasobarbus sublimus*, paratype (CMNFI 95-0011) from Rūdkhāneh-ye A‘lā, photo S. Tränkner.

**Figure 27.**
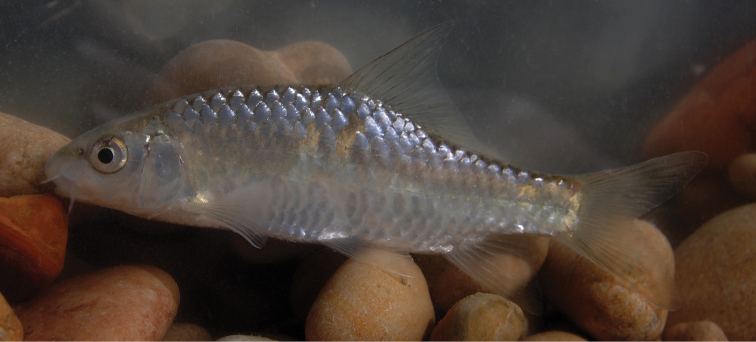
*Carasobarbus sublimus*, live specimen from Rūdkhāneh-ye Fahlīān.

A nuchal hump is not developed. The maximum body depth is at the anterior end of the dorsal fin base. The ventral profile of the head is almost straight; the dorsal profile is convex and evenly curved (Figs 26, 27). The maximum body depth is greater than the head length (Fig. 12). The mouth is inferior, narrow, the lips are thick and the lower jaw is spatulate with a horny sheath and a median lobe on the lower lip. The two pairs of barbels (Table 2) are well developed. The eyes are at the posterior end of the anterior half of the head. Some morphometric characters are summarised in Table 1.

The dorsal fin usually has four unbranched and nine or 10 branched rays (Table 3). The last unbranched ray of the dorsal fin is weakly ossified and about as long as the head (Fig. 4). The anal fin usually has three unbranched and six branched rays (Table 4) and its base is surrounded by a sheath of scales. Pectoral, ventral and anal fins are longer than in all other *Carasobarbus* species (Table 1).

There are 27 to 29 scales in the lateral line (Table 5), 4.5 or 5.5 scales above the lateral line (Table 6), 3.5 to 5.5 scales below the lateral line (Table 7) and 12 scales around the least circumference of the caudal peduncle (Table 8). The scales are shown in Fig. 5.

The pharyngeal teeth count is 2.3.4-5.3.2, 2.3.4-5.3.1 or 3.3.4-4.3.3 (Coad and Najafpour 1997). The pharyngeal bones available were too small for photography but are very similar to those of *Carasobarbus kosswigi* (Fig. 6).

Live specimens from Rūdkhāneh-ye Fahlīān are silvery with hyaline fins (Fig. 27). Live specimens from Rūdkhāneh-ye A‘lā are silvery with a slightly darker back, the scales have dark pigments on their hind margin; pectoral, ventral and anal fins have a yellow to orange hue, which is most obvious with fins folded back; dorsal and caudal fins are grey or hyaline (Coad and Najafpour 1997). Ethanol-preserved specimens are yellowish brown with a somewhat darker back and juveniles have a dark spot on the sides of the caudal peduncle.

*Carasobarbus sublimus* differs from all congeners, except *Carasobarbus kosswigi*, by having a spatulate lower jaw with a median lobe on the lower lip vs. a crescent shaped lower jaw and a lower lip without median lobe. It differs from *Carasobarbus kosswigi* by having 27 to 29 scales in the lateral line vs. 32 to 38 and modally 12 scales around the least circumference of the caudal peduncle vs. 14 and by having a shorter and less ossified unbranched last dorsal-fin ray.

#### Distribution.

This species is known from Rūdkhāneh-ye A‘lā, Rūdkhāneh-ye Fahlīān and possibly Rūdkhāneh-ye Kashgān (see discussion) in south-western Iran (Fig. 7).

#### Habitats and biology.

*Carasobarbus sublimus* is adapted to streams with fast currents with water flowing over hard substrate (Coad and Najafpour 1997). The biggest specimen known has a SL of 115 mm (Coad and Najafpour 1997).

#### Conservation status.

Little is known about the conservation status of *Carasobarbus sublimus*, but because this species is dependent on fast-flowing water, it is probably impacted by the construction of dams.

#### Discussion.

*Carasobarbus sublimus* was described in the genus *Barbus* and aligned with *Carasobarbus apoensis*, *Carasobarbus canis*, *Carasobarbus chantrei*, *Carasobarbus exulatus*, *Carasobarbus kosswigi* and *Carasobarbus luteus* in the original description (Coad and Najafpour 1997). Coad recommends the use of the genus *Kosswigobarbus* for this species (Coad 2011). It was transferred to *Carasobarbus*, based on morphological characters and close genetic relationship (Borkenhagen et al. 2011).

Locality data for CMNFI 79-0277 is not beyond doubt, because this lot was mentioned as *Carasobarbus kosswigi* in the original description of *Carasobarbus sublimus* (Coad and Najafpour 1997). According to morphometric and meristic characters (scales in lateral line, above lateral line and around the least circumference of the caudal peduncle; length of dorsal, pectoral, ventral and anal fin) this specimen is within the range of *Carasobarbus sublimus* and outside the range of *Carasobarbus kosswigi*. It might be an aberrant specimen or it might have been accidentally swaped with CMNFI 1995-0010 (a specimen of similar size from the same locality as the types of *Carasobarbus sublimus*). We had no opportunity to examine CMNFI 1995-0010. Though we think it is unlikely that *Carasobarbus kosswigi* and *Carasobarbus sublimus* occur sympatrically, for the time being we consider it to be a possible record of *Carasobarbus sublimus* from the Rūdkhāneh-ye Kashgān.

### Hybrids

Two putative intergeneric hybrids of *Carasobarbus canis* with other cyprinids are known, one with *Capoeta damascina* (Valenciennes in Cuvier and Valenciennes 1842) and one with *Luciobarbus longiceps* (Valenciennes in Cuvier and Valenciennes 1842).

#### *Carasobarbus canis*×*Capoeta damascina*

The hybrids are intermediate in many morphometric and meristic characters (Mir et al. 1988). The head resembles that of *Capoeta damascina*, the mouth is more inferior than in *Carasobarbus canis* and the lips are thicker. The scales are larger than in *Capoeta damascina* and smaller than in *Carasobarbus canis* (Mir et al. 1988, Fig. 28). Oogonia and spermatogonia coexist in the gonads of both sexes and the development of the gametes is disturbed, thus the hybrids are sterile (Fishelson et al. 1996).

**Figure 28.**
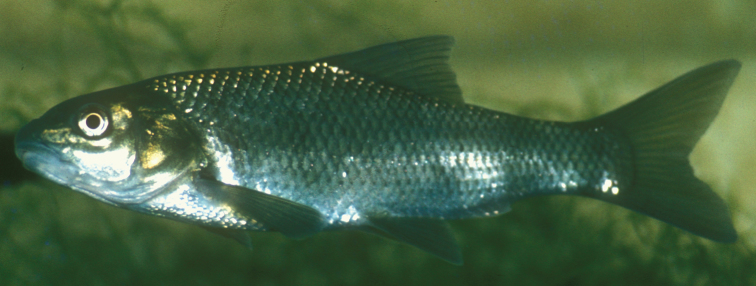
*Carasobarbus canis* x *Capoeta damascina*, aquarium photograph of SMF 17184, originally from Nahr az Zarqā’.

#### *Carasobarbus canis*×*Luciobarbus longiceps*

These hybrids are intermediate in many morphometric and meristic characters (Krupp 1985b, Fig. 29). The lateral line scale count matches that of *Carasobarbus canis*. Heterologous cells are present in the gonads of this hybrid but the gametes mature normally (Fishelson et al. 1996). This hybrid was described as *Barbus continii* Vinciguerra, 1926 from a single specimen (Krupp 1985b).

**Figure 29.**
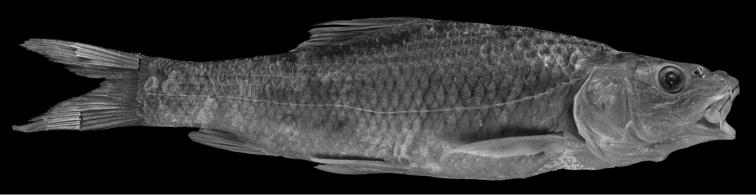
Holotype of *Barbus continii* = *Carasobarbus canis* x *Barbus longiceps* preserved specimen (SL=165 mm), Lake Tiberias (MCSN 22300).

### Key to the *Carasobarbus* species

**Table:** 

1	Branched dorsal-fin rays 9, Yemen and NW Africa	2
–	Branched dorsal fin rays 10	4
2	Scales around least circumference of the caudal peduncle 10−12, Yemen	*Carasobarbus exulatus*
–	Scales around least circumference of the caudal peduncle 13−20, Morocco	3
3	Dorsal profile of head convex, more than 15 % of the last unbranched dorsal-fin ray flexible	*Carasobarbus fritschii*
–	Dorsal profile of head straight, less than 15 % of the last unbranched dorsal-fin ray flexible	*Carasobarbus harterti*
4	Lower jaw spatulate and lower lip with a median lobe	5
–	Lower jaw u-shaped or crescent shaped and lower lip without median lobe	6
5	Scales around the least circumference of the caudal peduncle 12, 27−29 scales in the lateral line, head about as long as dorsal fin	*Carasobarbus sublimus*
–	Scales around the least circumference of the caudal peduncle 12−16, 32−38 scales in the lateral line, dorsal fin longer than the head	*Carasobarbus kosswigi*
6	Modally 14 (12−16) scales around the least circumference of the caudal peduncle	*Carasobarbus chantrei*
–	Modally 12 (10−14) scales around the least circumference of the caudal peduncle	7
7	Usually two pairs of barbels, Jordan River and adjacent waterbodies	*Carasobarbus canis*
–	Usually one pair of barbels, Mesopotamia, southern Iran and Arabia	8
8	Head about as long as body depth, dorsal fin markedly shorter than head, modally 30 scales in the lateral line, Western Arabian Peninsula	*Carasobarbus apoensis*
–	Head shorter than body depth, dorsal fin about as long as head (except in Iranian populations), modally 28 scales in lateral line, Mesopotamia and southern Iran	*Carasobarbus luteus*

## Authors contribution

KB and FK developed the concept for this study and conducted field research independent of each other. KB collected, analysed and interpreted the data presented in this study and prepared the manuscript. FK reviewed the manuscript.

## Supplementary Material

XML Treatment for Carasobarbus

XML Treatment for Carasobarbus apoensis

XML Treatment for Carasobarbus canis

XML Treatment for Carasobarbus chantrei

XML Treatment for Carasobarbus exulatus

XML Treatment for Carasobarbus fritschii

XML Treatment for Carasobarbus harterti

XML Treatment for Carasobarbus kosswigi

XML Treatment for Carasobarbus luteus

XML Treatment for Carasobarbus sublimus

## Appendix

Table of localities for all lots examined. (doi: 10.3897/zookeys.339.4903.app) File format: OpenDocument spreadsheet (ods).

**Explanation note:** Table of localities for all specimens examined as a spreadsheet to make them more easily available for use in biodiversity databases and geospatial investigations.
